# Cediranib in patients with alveolar soft-part sarcoma (CASPS): a double-blind, placebo-controlled, randomised, phase 2 trial

**DOI:** 10.1016/S1470-2045(19)30215-3

**Published:** 2019-07

**Authors:** Ian Judson, James P Morden, Lucy Kilburn, Michael Leahy, Charlotte Benson, Vivek Bhadri, Quentin Campbell-Hewson, Ricardo Cubedo, Adam Dangoor, Lisa Fox, Ivo Hennig, Katy Jarman, Warren Joubert, Sarah Kernaghan, Antonio López Pousa, Catriona McNeil, Beatrice Seddon, Claire Snowdon, Martin Tattersall, Christy Toms, Javier Martinez Trufero, Judith M Bliss

**Affiliations:** a The Institute of Cancer Research and The Royal Marsden NHS Foundation Trust, London, UK; b Clinical Trials and Statistics Unit, The Institute of Cancer Research, London, UK; c The Christie NHS Foundation Trust, Manchester, UK; d The Royal Marsden NHS Foundation Trust, London, UK; e Chris O'Brien Lifehouse, Sydney, NSW, Australia; f Newcastle upon Tyne Hospitals NHS Foundation Trust, Newcastle upon Tyne, UK; g Hospital Puerta de Hierro, Madrid, Spain; h University Hospitals Bristol NHS Foundation Trust, Bristol, UK; i Nottingham University Hospitals NHS Trust, Nottingham, UK; j Princess Alexandra Hospital, Brisbane, QLD, Australia; k Hospital de la Santa Creu i Sant Pau, Barcelona, Spain; l University College London Hospitals NHS Foundation Trust, London, UK; m Hospital Miguel Servet, Zaragoza, Spain

## Abstract

**Background:**

Alveolar soft-part sarcoma (ASPS) is a rare soft-tissue sarcoma that is unresponsive to chemotherapy. Cediranib, a tyrosine-kinase inhibitor, has shown substantial activity in ASPS in non-randomised studies. The Cediranib in Alveolar Soft Part Sarcoma (CASPS) study was designed to discriminate the effect of cediranib from the intrinsically indolent nature of ASPS.

**Methods:**

In this double-blind, placebo-controlled, randomised, phase 2 trial, we recruited participants from 12 hospitals in the UK (n=7), Spain (n=3), and Australia (n=2). Patients were eligible if they were aged 16 years or older; metastatic ASPS that had progressed in the previous 6 months; had an ECOG performance status of 0–1; life expectancy of more than 12 weeks; and adequate bone marrow, hepatic, and renal function. Participants had to have no anti-cancer treatment within 4 weeks before trial entry, with exception of palliative radiotherapy. Participants were randomly assigned (2:1), with allocation by use of computer-generated random permuted blocks of six, to either cediranib (30 mg orally, once daily) or matching placebo tablets for 24 weeks. Treatment was supplied in number-coded bottles, masking participants and clinicians to assignment. Participants were unblinded at week 24 or sooner if they had progression defined by Response Evaluation Criteria in Solid Tumors (version 1.1); those on placebo crossed over to cediranib and all participants continued on treatment until progression or death. The primary endpoint was percentage change in sum of target marker lesion diameters between baseline and week 24 or progression if sooner, assessed in the evaluable population (all randomly assigned participants who had a scan at week 24 [or sooner if they progressed] with target marker lesions measured). Safety was assessed in all participants who received at least one dose of study drug. This study is registered with ClinicalTrials.gov, number NCT01337401; the European Clinical Trials database, number EudraCT2010-021163-33; and the ISRCTN registry, number ISRCTN63733470 recruitment is complete and follow-up is ongoing.

**Findings:**

Between July 15, 2011, and July 29, 2016, of 48 participants recruited, all were randomly assigned to cediranib (n=32) or placebo (n=16). 23 (48%) were female and the median age was 31 years (IQR 27–45). Median follow-up was 34·3 months (IQR 23·7–55·6) at the time of data cutoff for these analyses (April 11, 2018). Four participants in the cediranib group were not evaluable for the primary endpoint (one did not start treatment, and three did not have their scan at 24 weeks). Median percentage change in sum of target marker lesion diameters for the evaluable population was −8·3% (IQR −26·5 to 5·9) with cediranib versus 13·4% (IQR 1·1 to 21·3) with placebo (one-sided p=0·0010). The most common grade 3 adverse events on (blinded) cediranib were hypertension (six [19%] of 31) and diarrhoea (two [6%]). 15 serious adverse reactions in 12 patients were reported; 12 of these reactions occurred on open-label cediranib, and the most common symptoms were dehydration (n=2), vomiting (n=2), and proteinuria (n=2). One probable treatment-related death (intracranial haemorrhage) occurred 41 days after starting open-label cediranib in a patient who was assigned to placebo in the masked phase.

**Interpretation:**

Given the high incidence of metastatic disease and poor long-term prognosis of ASPS, together with the lack of efficacy of conventional chemotherapy, our finding of significant clinical activity with cediranib in this disease is an important step towards the goal of long-term disease control for these young patients. Future clinical trials in ASPS are also likely to involve immune checkpoint inhibitors.

**Funding:**

Cancer Research UK and AstraZeneca.

## Introduction

Alveolar soft-part sarcoma (ASPS) is rare, accounting for less than 0·5% of all soft-tissue sarcomas. It predominantly affects young people, with a median age at presentation of 25 years and most patients younger than 30 years at diagnosis.^1^ ASPS commonly involves the lower limb, with a slight predominance in women and a high incidence of metastatic disease at diagnosis.^1^ Although metastases are intrinsically indolent, the long-term outlook is poor.^2^ Lieberman and colleagues^2^ report that only 15% of patients with no metastases at diagnosis remained metastasis free after 20 years of follow-up, with a median metastasis-free period of 6 years and median survival after development of metastases of 2 years. If patients presented with metastases, median survival was 3 years, compared with 11 years for patients who were metastasis free at diagnosis, and survival tended to worsen with increasing age.^2^ Unusually for a soft-tissue sarcoma, in addition to lung metastases, ASPS also metastasises to brain and bone.^3^ Histologically, the disease is characterised by uniform polygonal cells arranged in a pseudoalveolar pattern separated by vascular septae, and molecular studies^4^ have shown a characteristic non-reciprocal translocation, t(X;17)(p11·2;q25), resulting in the *ASPSCR1–TFE3* fusion gene that replaces the N-terminal portion of *TFE3* in a manner consistent with transcriptional deregulation.^4^

**Research in context**

**Evidence before this study**

Before undertaking this study, the available data concerning the activity of the experimental drug cediranib (previously AZD2171) in the treatment of alveolar soft part sarcoma (ASPS) consisted of an index case of extended response in ASPS in a phase 2 trial, a phase 2 study of cediranib in the treatment of gastrointestinal stromal tumours and soft tissue sarcomas including ASPS, and a phase 2 single-arm study, which was ongoing at the time the CASPS trial was being developed and has since shown activity in ASPS. Other studies involving ASPS, ongoing and completed, were identified using the ClinicalTrials.gov website, searching for “alveolar soft part sarcoma”; PubMed, searching for publications in English between Jan 1, 2000, and Dec 31, 2018, using the search terms “tyrosine kinase inhibitor”, “anti-angiogenic agent”, and “alveolar soft part sarcoma”; via presentations at international meetings, and personal communications. Other drugs with reported activity in ASPS include sunitinib, pazopanib, and anlotinib.

**Added value of this study**

ASPS has a high metastatic potential, but usually slow disease progression, and sometimes spontaneous disease arrest and even, rarely, regression can occur. These characteristics make progression-free survival an unreliable endpoint for this disease. By undertaking a placebo-controlled randomised trial with tumour size as the primary endpoint, we ensured that the activity of cediranib could be measured reliably. To our knowledge, this study is the only randomised trial to be reported in ASPS so far.

**Implications of all the available evidence**

Although the precise molecular targets of cediranib in ASPS are not known, this study has confirmed the value of this drug in the treatment of advanced ASPS. The relative importance of angiogenesis inhibition and immunomodulation are the subject of active investigation and translational research from this study will be published in due course. Confirmation of the activity of cediranib in a randomised, placebo-controlled trial will provide a sound basis for future research with this and other drugs for ASPS.

ASPS cells have periodic acid-Schiff (PAS)-positive precrystalline granules that contain monocarboxylate transporter 1 (MCT1) and its chaperone basigin (CD147).^5^ In a genetically engineered mouse study^6^ that used the *ASPSCR1–TFE3* gene to drive oncogenesis, the mice developed tumours in the brain and orbit—ie, the cranial vault—a region known to have the highest lactate concentrations in the mouse. Metabolic studies^6^ showed that ASPS cells in this model used lactate as an energy source. Lactate is imported via MCT1 and is converted directly to pyruvate for entry into the citric acid cycle. In addition to supplying energy, lactate acts to stimulate cell proliferation and angiogenesis because of the excess pyruvate, which upregulates hypoxia-inducible factor 1-α (HIF1-α) via inhibition of the prolyl hydroxylase responsible for its degradation,^7^ raising the possibility that the lactate transporter MCT1 could be a therapeutic target for inhibition of tumour angiogenesis.^8^

Cediranib is a receptor tyrosine-kinase inhibitor, the targets of which include the VEGF receptors VEGFR1, VEGFR2, and VEGFR3; KIT; and platelet-derived growth factor receptors. After observation of a prolonged partial remission with cediranib in a patient with locally advanced and metastatic ASPS treated in a phase 2, hypertension management study (NCT00264004),^9^ an opportunity arose to treat a further six patients with ASPS who were treated in a cediranib pharmacodynamic study of patients with soft-tissue sarcoma, most of whom had gastrointestinal stromal tumours (D8480C00046).^10^ Strong evidence of the clinical activity of cediranib against ASPS, in terms of durable partial remissions and disease stabilisation, led to further studies including a single-arm, phase 2 trial of cediranib in ASPS by the US National Cancer Institute.^11^

Other tyrosine-kinase inhibitors have also been shown to have activity in ASPS. A direct antitumour effect has been shown with sunitinib mediated by platelet-derived growth factor receptor β (PDGFRB), VEGFR2, and RET, with five partial responses in nine patients and median progression-free survival on treatment of 17 months (range 2–33).^12^ In adults, a retrospective study reported one complete response and seven partial responses in 30 patients treated with pazopanib, with a median progression-free survival of 13·6 months (range 1·6–32·2) and only little activity with trabectedin.^13^ In 40 patients with ASPS and a rearrangement of the *TFE3* gene treated with the serine/threonine-protein kinase Haspin homolog ALK1 and hepatocyte growth factor receptor (MET) inhibitor crizotinib, one patient had a partial response and 35 had stable disease as their best response, with 1-year progression-free survival of 37·5% (95% CI 22·9–52·1).^14^ Anlotinib has also been reported to have activity in a prospective basket study^15^ in which six of 13 patients with ASPS had partial responses and the median progression-free survival was 21 months. Pazopanib is the only multi-targeted tyrosine-kinase inhibitor approved for second-line or further-line treatment of all soft-tissue sarcomas. Preliminary reports of activity with immunomodulatory drugs have been presented in the past 2 years.^15^, ^16^

The Cediranib in Alveolar Soft Part Sarcoma (CASPS) trial aimed to assess the efficacy of cediranib in the treatment of ASPS. The double-blind, placebo-controlled design was chosen because of the unusual biology of this cancer, which, although having strong metastatic potential, is characterised by indolent metastatic tumour growth and periods of spontaneous stabilisation, making single-group uncontrolled studies difficult to interpret.^3^, ^12^ Ethical challenges exist in placebo groups, which we mitigated by having a 2:1 randomisation favouring active treatment, restricting the no-treatment period to 24 weeks, and allowing crossover to active treatment on disease progression or, if no progression, after week 24. At entry, patients were required to have progressed in the previous 6 months, hence 6 months was chosen as the period for comparison between cediranib and placebo. A preliminary report of CASPS was presented at the 2017 Annual General Meeting of the American Society of Clinical Oncology.^17^

## Methods

### Study design and participants

In this multicentre, double-blind, placebo-controlled, randomised, phase 2 trial, participants were recruited from 12 hospitals in the UK (n=7), Spain (n=3), Australia (n=2). Eligible patients were women or men aged 16 years or older, with a histologically confirmed diagnosis of ASPS. A tumour block was required for central review and confirmation of the presence of t(X;17)(p11·2:q25) translocation. Patients were required to have measurable metastatic disease that had progressed according to the Response Evaluation Criteria in Solid Tumors (RECIST) version 1.1 in the previous 6 months; an ECOG performance status of 0–1; life expectancy of more than 12 weeks; and adequate bone marrow, hepatic, and renal function (absolute neutrophil count >1·5 × 10^9^ per L; platelet count >100×10^9^ per L; serum bilirubin <1·5 × upper limit of normal [ULN], unless proven Gilbert's syndrome; alanine transaminase or aspartate transaminase <2·5 × ULN or <5 × ULN if liver metastases present; serum creatinine <1·5 × ULN or creatinine clearance >50 mL per min). The restrictive criterion of progression in the previous 6 months was based on the known indolent nature of the disease. CASPS thus differs from other ASPS studies that do not have such restricted eligibility criteria. Patients with brain metastases were eligible if their disease was controlled with a stable dose of corticosteroid or a non-enzyme-inducing anticonvulsant. Exclusion criteria included history of gastrointestinal disorder likely to impair absorption of cediranib; poorly controlled hypertension; any severe or uncontrolled comorbidity—eg, active infection; prolonged QT interval—ie, QTc ≥480 msec (using Bazetts correction) or family history of long QT syndrome; substantial recent haemorrhage (>30 mL bleeding or episode in previous 3 months); major thoracic or abdominal surgery in previous 14 days; recent history of thrombosis; pregnant or breastfeeding women; unwillingness to use adequate birth control measures; anticancer treatment in the previous 4 weeks, with the exception of palliative radiotherapy; known hypersensitivity to any excipient of cediranib; history of other malignancy except cancer in situ unless individual had been disease free for more than 2 years and with tissue diagnosis of ASPS from target lesion; and any other concomitant anticancer therapy except steroids. A full list of exclusion criteria is in the appendix (pp 27–28). Previous treatment with cediranib was added as an exclusion criterion and approved as a protocol amendment on the advice of the joint Independent Data Monitoring and Steering Committee (IDMSC) on Sept 24, 2014.

Patients provided written, informed consent before enrolment. The study protocol is in the appendix (pp 9–75). The study was approved by the South West London Research Ethics Committee 4 (REC reference 10/H0806/118) in the UK; the Clinical Research Ethics Committee of Hospital de la Santa Creu i Sant Pau, Barcelona (12/070 [R]), in Spain; and by the Metro South Health Service District Human Research Ethics Committee, QLD (HREC/12/QPAH/10), and the Sydney Local Health District Human Research Ethics Committee (HREC/12/RPAH/26), in Australia. The Clinical Trials and Statistics Unit at The Institute of Cancer Research, London, UK (ICR-CTSU), had overall responsibility for trial coordination with two international trials groups (Grupo Español de Investigación en Sarcomas [GEIS], Madrid, Spain, and Australasian Sarcoma Study Group [ASSG], Melbourne, Australia) having responsibility for regulatory and ethics submissions, monitoring, and safety reporting within their respective countries. Safety and efficacy data were reviewed regularly by the IDMSC. A Trial Management Group was responsible for the day-to-day running of the trial. The ICR-CTSU undertook all central statistical monitoring, interim, and final analyses.

### Randomisation and masking

Participants were randomly allocated (2:1) to cediranib or placebo by computer-generated random permuted block method (block size of six) derived by ICR-CTSU, and the randomisation sequence centrally was generated centrally at the ICR-CTSU. Because of the small trial size, we did not use any stratification factors. Both the participants and clinicians were masked to treatment allocation until week 24 or until disease progression if this occurred sooner. Treatment was supplied in number-coded bottles, masking participants and clinicians to assignment.

### Procedures

Depending on treatment allocation, participants received cediranib or matching placebo 30 mg orally once daily, for the first 24 weeks of the study. At 24 weeks, or sooner if the patient had confirmed disease progression according to RECIST, participants were unmasked. Participants allocated to placebo crossed over to open-label cediranib and continued on treatment until disease progression or death. Participants allocated to cediranib who had not progressed by 24 weeks continued on cediranib until progression or death. Participants could withdraw from the trial at any point and for any reason.

Clinical assessments, including physical examination, symptom review, and routine blood and urine investigations, took place at 2 and 4 weeks, then once every 4 weeks until week 24, every 8 weeks up to 48 weeks, then every 12 weeks until progression or treatment discontinuation. Assessments for participants crossing over from placebo to cediranib were recommenced similarly to in the first 24 weeks for the original cediranib group. Tumour assessments (CT or MRI if indicated) were done at baseline, every 8 weeks until week 48, and then every 12 weeks until disease progression or death. Blood pressure was monitored at least weekly for the first 4 weeks, then monthly up to 24 weeks and thereafter as specified in the protocol—ie, every 8 weeks up to 48 weeks and every 12 weeks thereafter. Masked radiology review was not planned as part of this study, although translational imaging studies are planned.

Toxicity was assessed using National Cancer Institute Common Terminology Criteria for Adverse Events (CTCAE) version 4 on the same schedule as the clinical assessments. Coding was done by use of the Medical Dictionary for Regulatory Activities (version 14).

Treatment-related toxicities of grade 3 or worse, or repeated episodes of grade 2 toxicities not responding to adequate supportive measures were managed initially with dose interruptions of 2–5 days, with reintroduction on resolution at the same dose. Longer interruptions up to 14 days were permitted for chronic problems such as nausea, diarrhoea, and palmar-plantar erythrodysaesthesia syndrome if refractory to supportive treatment—eg, antiemetics or loperamide. If toxic effects continued, a dose reduction to 20 mg daily was permitted. Treatment with cediranib was to be discontinued permanently for gastrointestinal perforation, wound dehiscence, severe haemorrhage, or severe uncontrolled hypertension. Abnormal thyroid function was treated with L-thyronine as appropriate.

### Outcomes

The primary endpoint was percentage change in the sum of the longest diameters (or shortest if nodal disease) of target marker lesions, measured at 24 weeks (or at progression if sooner) from the date of treatment assignment. Protocol-defined secondary endpoints were the proportion of participants with an objective response at week 24, defined as the proportion of participants having either a partial or complete response at the end of blinded treatment; best response up to week 24, defined as best response at any point during blinded treatment; best percentage reduction of target marker lesion size during blinded treatment; progression-free survival defined as time from random assignment to disease progression (defined by RECIST version 1.1) or death from any cause; the proportion of patients alive and progression free at 12 months; overall survival, defined as time from random assignment to death (any cause); and the safety and tolerability profile of cediranib in all participants. For progression free survival, participants who were alive and progression-free were censored at the date of last known follow-up. Participants with non-RECIST confirmed progression (eg, radiologically confirmed but lesions not measured according to RECIST, or participants with clinical evidence of progression only) were censored at the date of their progression.

Exploratory endpoints included radiological responses using Choi criteria, identification of predictive angiogenesis markers of response, to describe changes in angiogenesis markers and expression of angiogenesis regulatory genes in peripheral blood and optional tumour biopsies, and to explore changes in circulating endothelial cells and other rare cells events, including potential sarcoma circulating cells. These exploratory endpoints will be reported elsewhere once these data are available.

### Statistical analysis

Our study size was informed by previous studies of participants with metastatic ASPS. In the previously mentioned cediranib pharmacodynamic phase 2 study (D8480C00046),^10^ of 36 participants (ten of whom had soft-tissue sarcoma and 26 had a gastrointestinal stromal tumour), six participants with metastatic ASPS had a mean decrease in tumour size at 8 weeks of 25%, with a coefficient of variation of 19%. The second scan at 16 weeks showed that four of six participants had a more than 30% decrease in tumour size—ie, a partial response. We assumed that a smaller effect and greater coefficient of variation than these results might be observed in a larger trial; therefore, we determined that 36 participants would be required to detect a 20% decrease in the sum of the diameters of target marker lesions at 24 weeks between placebo and cediranib with a coefficient of variation of 25%, 80% power, and a one-sided significance level of 5%. We calculated this sample size for a two-sample *t* test of the log transformation of the sum of the diameters of target marker lesions using the sampsi command in Stata. We did a formal interim analysis after 18 participants (12 assigned to cediranib and six assigned to placebo) had 24 weeks of follow-up data (or had disease progression if sooner) to determine early activity of cediranib. Recruitment to the study was not halted while the interim analysis was undertaken.

We used descriptive statistics to show the baseline demographic information of the participants who were enrolled. We compared the primary endpoint between cediranib and placebo groups using the Wilcoxon-Mann-Whitney test. We chose to present the data as medians and IQRs and to use non-parametric tests a priori because of the uncertainty around whether or not the data would be normally distributed. We used waterfall plots to graphically present the primary endpoint result. The principal analysis population for the primary endpoint was the evaluable population, defined as all randomly assigned participants (the intention-to-treat [ITT] population) who had a scan at week 24 (or a scan at progression if earlier) with target marker lesions measured. We did a sensitivity analysis of the primary endpoint and of the best percentage change in the sum of the longest diameters of target marker lesions at 24 weeks based on this population, but excluding two participants who had received cediranib before entering the study (an exclusion criterion added after these two participants were enrolled).

We report binary endpoints (objective response, best response, and clinical benefit) as proportions and compared them between treatment groups using Fisher's exact test (one sided). The population analysed for best response also included participants who had a scan before 24 weeks, but were not evaluable for the primary endpoint. For survival-related endpoints, we used the ITT population; thus, we still analysed participants allocated to placebo who subsequently crossed over as belonging to the placebo group. We plotted Kaplan-Meier curves for the two treatment groups and compared them with the log-rank test. We calculated hazard ratios (HRs) and 90% CIs from Cox proportional hazards regression models for progression-free survival, with HRs of less than 1 favouring cediranib. HRs were not calculated for overall survival because of the failure of the proportional hazards assumption that is required to use Cox regression, as anticipated before the analysis. All analyses were unadjusted. Similar to the primary endpoint, we did a sensitivity analysis for progression-free survival and overall survival, excluding two participants who had received cediranib before entering the study. We planned two additional sensitivity analyses for progression-free survival: one to include participants who had non-RECIST confirmed progression, and the other was a landmark analysis, also including participants who had non-RECIST confirmed progression but censoring participants 26 weeks after random assignment to provide insight into progression-free survival in the absence of crossover.

The safety analysis population included all treated participants (ie, all those who received at least one dose of study drug or placebo), and we summarise the worst grades of adverse events during the blinded treatment phase. We did no formal comparisons of safety between treatment groups because of the small patient numbers. We present here any adverse event that was reported in at least 10% of participants in either treatment group, or in one or more participants for grade 3 and worse events. We also report on the number of serious adverse reactions to cediranib.

We also did an unplanned, exploratory analysis to explore the effect of previous tyrosine-kinase inhibitor therapy on progression-free survival.

Post hoc, we analysed duration of response (calculated as time from first response, complete or partial response, to date of progression), and the proportion of participants in each group who had a clinical benefit at 24 weeks for completeness and to enable comparison with other studies.

Analyses are based on a database snapshot taken on April 11, 2018. We did all analyses with Stata (version 13.1). This study is registered with ClinicalTrials.gov, number NCT0133740; on the ISRCTN registry, number ISRCTN 63733470; and on the European Clinical Trials database, number EudraCT 2010-021163-33.

### Role of the funding sources

The funders of the study had no role in study design, data collection, data analysis, data interpretation, or writing of the report. The corresponding author had full access to all data in the study and final responsibility for the decision to submit for publication.

## Results

**Figure 1:**
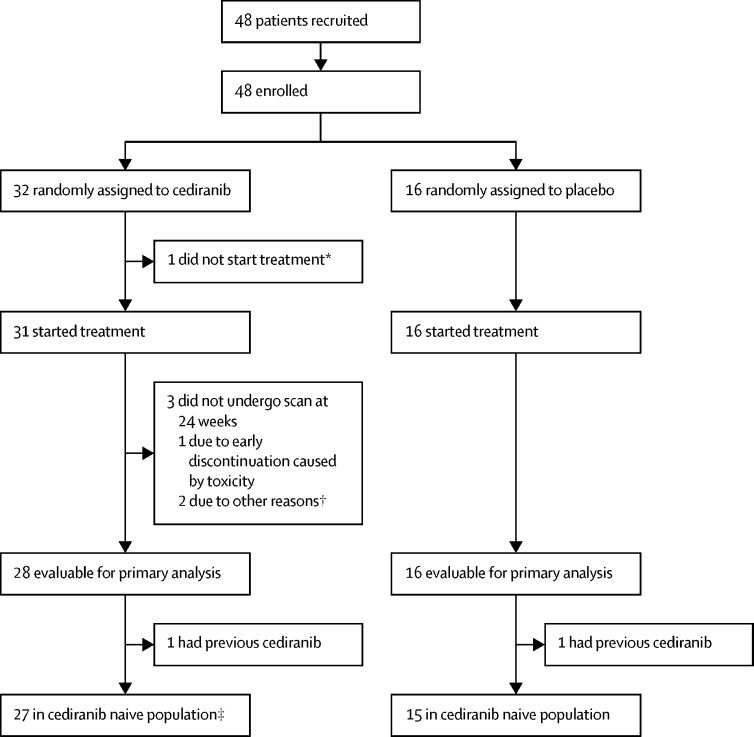
Trial profile *Found to have a tumour infiltrating their heart after random assignment. †One patient was subsequently found to be ineligible because of unconfirmed progression in the 6 months before trial entry. ‡One patient was subsequently found to be ineligible because of unconfirmed progression in the 6 months before trial entry, but was included in analyses.

Between July 15, 2011, and July 29, 2016, 48 patients recruited from 12 hospitals in three countries (n=31 from seven hospitals in the UK, n=9 from three hospitals in Spain, and n=8 from two hospitals in Australia) were enrolled. More participants were recruited than were originally planned under the recommendation of the IDMSC to account for the four participants who were not evaluable for the primary endpoint and two participants who had received cediranib previously. 32 participants were randomly assigned to cediranib and 16 to placebo (figure 1) and baseline characteristics were well balanced between the treatment groups (table 1). Median age at baseline was 31 years (IQR 27–45), with most participants in the 20–29 years age group. More than half of participants were diagnosed within 2·5 years of trial entry, the most common site of disease was the upper leg or groin and 11 (23%) of 48 participants had known brain metastases at trial entry. 20 (42%) participants had previously received tyrosine-kinase inhibitor treatment, including two (4%) with previous cediranib.

**Table 1:** Baseline demographic and clinical characteristics and previous treatments received for ASPS prior to trial entry

			**Cediranib group (n=32)**	**Placebo group (n=16)**
**Sex**				
Male			17 (53%)	8 (50%)
Female			15 (47%)	8 (50%)
**Age at baseline, years**				
Median			30·3 (26·8–44·7)	33·1 (29·3–43·5)
<20			0	1 (6%)
20–29			15 (47%)	5 (31%)
30–39			8 (25%)	4 (25%)
40–49			4 (13%)	5 (31%)
50–59			4 (13%)	0
60–69			0	1 (6%)
≥70			1 (3%)	0
**ECOG performance status**				
0			16 (50%)	9 (56%)
1			16 (50%)	7 (44%)
**Time since original ASPS diagnosis, years**				
≤2·5			18 (56%)	8 (50%)
>2·5–5			4 (13%)	6 (38%)
>5–7·5			2 (6%)	0
>7·5–10			2 (6%)	0
>10			6 (19%)	2 (13%)
**Site of primary disease**				
Upper leg or groin			14 (44%)	7 (44%)
Upper limb			6 (19%)	2 (13%)
Lower leg or foot			3 (9%)	3 (19%)
Trunk			4 (13%)	2 (13%)
Buttock			3 (9%)	0
Pelvis			1 (3%)	2 (13%)
Cranial or facial			1 (3%)	0
**Synchronous metastases at trial entry**				
Yes			5 (16%)	0
No			27 (84%)	16 100%)
**Brain metastases at trial entry**				
Yes			8 (25%)	3 (19%)
No			24 (75%)	13 (81%)
**Previous treatment for ASPS**				
Any localised treatment				
	No		7 (22%)	5 (31%)
	Yes		25 (78%)	11 (69%)
		Surgery	21 (66%)	11 (69%)
		To the primary disease site	19 (59%)	10 (63%)
		For metastatic disease	10 (31%)	4 (25%)
	Radiotherapy		21 (66%)	7 (44%)
		To the primary disease site	12 (38%)	7 (44%)
**Any systemic treatment**				
No			19 (59%)	7 (44%)
Yes			13 (41%)	9 (56%)
	Chemotherapy		5 (16%)	4 (25%)
	Tyrosine-kinase inhibitor		12 (38%)	8 (50%)
		Crizotinib	5 (16%)	5 (31%)
		Sunitinib	1 (3%)	2 (13%)
		Axitinib	2 (6%)	0
		Pazopanib	2 (6%)	0
		Cediranib	1 (3%)	0
		Cediranib plus dovitinib	0	1 (6%)
		Sunitinib plus pazopanib	1 (3%)	0
	MET inhibitor (ARQ197)		1 (3%)	0
	HDAC inhibitor (PXD101)		0	1 (6%)

Data are n (%) or median (IQR). ASPS=alveolar soft part sarcoma. MET=hepatocyte growth factor receptor. HDAC=histone deacetylase.

A planned interim analysis was done of a snapshot of data taken on Feb 4, 2014, and did not meet the stopping criteria for the primary endpoint (appendix p 57), thus the study continued until the primary analysis. Of 32 participants randomly assigned to cediranib, one (3%) did not start treatment and three (6%) did not have their scan at 24 weeks (one of whom discontinued due to toxicity). Median time on blinded treatment was 23·9 weeks (IQR 20·1–24·6) for participants assigned to cediranib and 22·2 weeks (IQR 9·6–23·8) for those assigned to placebo. Of those who started treatment, 14 (45%) in the cediranib group and four (25%) in the placebo group had at least one dose modification, dose delay, or missed dose during blinded treatment because of adverse events. 11 (35%) of 31 who were given blinded cediranib did not continue to open-label treatment; eight (26%) because of disease progression, one (3%) because of haematemesis, one (3%) because of palmar-plantar erythrodysesthesia and fatigue, and one (3%) because of general poor tolerance and planned surgery. All participants allocated to placebo went on to have at least one dose of open-label cediranib. Of 36 (75%) participants who started open-label cediranib, eight (five from the cediranib group and three from the placebo group) were still on treatment at the data cutoff for this analysis. Reasons for discontinuation in the remaining 28 participants were disease progression (n=21), adverse events (n=2), patient choice (n=4), and death (n=1). Median time on open-label cediranib was 41·6 weeks (IQR 21·0–67·0) for the participants from the cediranib group and 40·3 weeks (12·2–108·6) for those from the placebo group.

**Figure 2:**
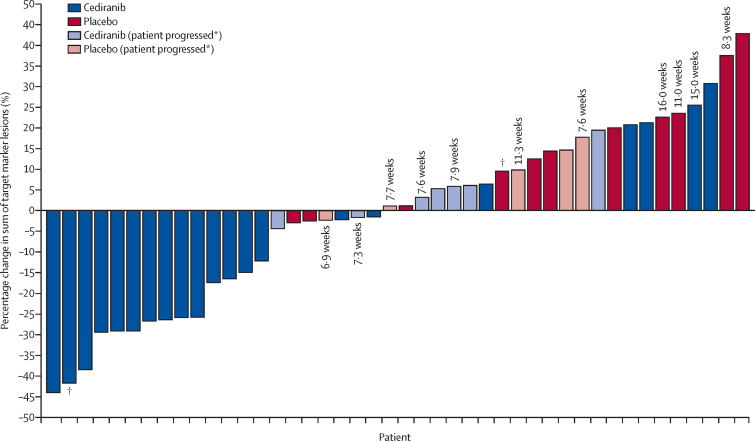
Percentage change in sum of target marker lesions from baseline to week 24 (or progression if sooner) in all evaluable participants (n=44) Each bar represents one patient. Where the number of weeks is given, it indicates the timepoint at which progression occurred for those who did not reach the 24 week assessment. *Patients who progressed had either progression of non-target lesions or appearance of new lesions despite a less than 20% decrease in the sum of target marker lesions. †Patient received cediranib before trial entry.

In the evaluable population (44 [92%] of 48 enrolled patients), we found a significant difference in the median percentage change in the sum of the diameters of target marker lesions at 24 weeks of −8·3% (IQR −26·5 to 5·9) in the cediranib group compared with 13·4% (1·1 to 21·3) in the placebo group (one-sided p=0·0010; figure 2).

Best median percentage change in the sum of the diameters of target marker lesions by 24 weeks was −15·7% (IQR −26·3 to −2·4) with cediranib and 1·2% (−2·4 to 10·9) with placebo (one-sided p<0·0001; appendix p 2).

Similar results were obtained in the sensitivity analysis in the cediranib-naive population (42 [88%] of 48 participants): median percentage change in the sum of the longest diameters (or shortest if nodal disease) of target marker lesions at 24 weeks was −4·4% (IQR −26·3 to 6·0) with cediranib versus 14·4% (1·1 to 22·6) with placebo (one-sided p=0·0019) and best percentage change was −15·0% (IQR −26·3 to −2·4) with cediranib versus 1·3% (−2·5 to 11·8) with placebo (one-sided p=0·00010).

The proportion of participants with an objective response, based on best response during the blinded treatment phase, was 19%, with six of 31 participants on cediranib having a partial response at any timepoint during the masked treatment phase. However, at week 24, three of these participants no longer met RECIST criteria for partial response, and thus the proportion of participants with an objective response at week 24 was 11% (three of 28). No participants in the placebo group had a partial response during the blinded treatment phase (one-sided p=0·072, cediranib *vs* placebo). 14 (50%) of 28 participants in the cediranib group and seven (44%) of 16 in the placebo group had stable disease at 24 weeks; thus the proportion of participants with clinical benefit at 24 weeks was 61% (17 of 28) for cediranib and 44% (seven of 16) for placebo (post-hoc analysis). Of the seven participants in the placebo group who were stable at 24 weeks, three had received no treatment in the 6 months before randomisation, one had surgery to the primary disease site, and another three were on another tyrosine-kinase inhibitor until a month before randomisation. Of the 14 patients randomly assigned to cediranib with stable disease or better at 24 weeks before therapy, three had no previous treatment; eight had local therapy (surgery or radiotherapy, or both) only to primary or metastatic sites, or both; two had local therapy and another tyrosine-kinase inhibitor; and one had another tyrosine-kinase inhibitor only. Median duration of response in the six patients in the cediranib group who had a partial response during the blinded treatment phase was 16·0 months (IQR 15·7–26·0); all six patients have subsequently progressed (post hoc). Of the 32 participants assigned to the cediranib group, two (6%) who had stable disease up to week 24 subsequently achieved a partial response (one by week 32, the other by week 40). Notably, at the time of primary analysis with a minimum of 6 months follow-up after random assignment to treatment, four (25%) of 16 participants in the placebo group had not progressed. Percentage change in the sum of the diameters of target marker lesions from randomisation to beyond week 24 is shown in the appendix (pp 3–4).

**Figure 3:**
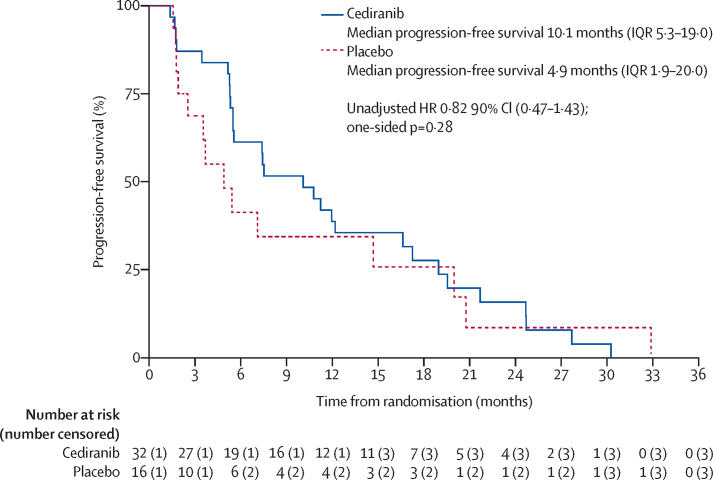
Progression-free survival HR=hazard ratio.

**Figure 4:**
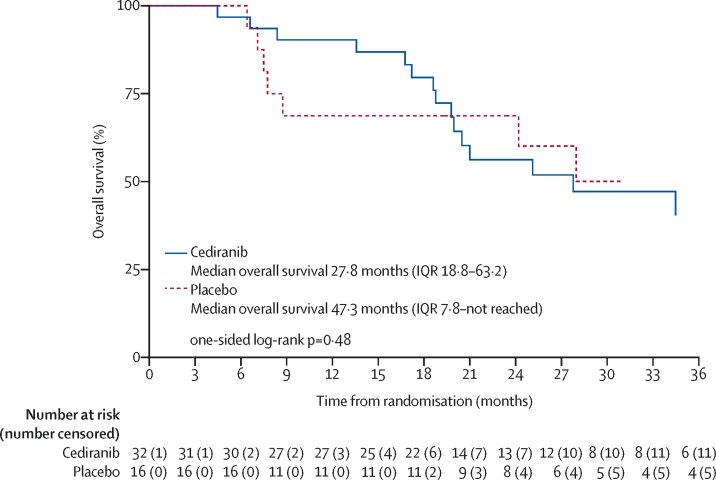
Overall survival Hazard ratio was not calculated for overall survival due to violation of the non-proportionality assumption.

At the time of data cutoff for this analysis (median follow-up of 34·3 months [IQR 23·7–55·6]), the follow-up period was dominated by time on open-label cediranib and 43 (90%) of 48 participants (29 from the cediranib group, 14 from the placebo group) had had RECIST-confirmed progression or death before progression. We found no evidence of a significant difference in progression-free survival between the treatment groups (unadjusted HR 0·82, 90% CI 0·47–1·43; p=0·28; figure 3), although, this analysis was probably confounded by the crossover to cediranib. 12-month progression-free survival was 38·7% (95% CI 22·0–55·2) for cediranib and 34·4% (12·7–57·5) for placebo. In a sensitivity analysis excluding those participants with previous cediranib treatment, a similar result was achieved (unadjusted HR 0·76, 90% CI 0·43–1·35; one sided p=0·21). We expect that with extended follow-up, the progression-free survival curves will increasingly converge due to the proportional effect of crossover to active therapy in those patients who were originally allocated to placebo. The first planned additional sensitivity analysis included three (6%) of 48 participants (one in the cediranib group and two in the placebo group) who had non-RECIST confirmed progression (appendix p 5). This analysis showed no evidence of a difference between the two groups (HR 0·73, 90% CI 0·43–1·24; one-sided p=0·16). The second planned sensitivity analysis, the landmark analysis, showed a significant improvement in progression-free survival for participants allocated to the cediranib group (appendix p 6). At the time of analysis, 24 (50%) of 48 participants had died (16 in the cediranib group and eight in the placebo group). As expected, we found no evidence of a difference in overall survival between the groups (figure 4). Overall survival estimates at 12 months were 90·3% (95% CI 72·9–96·8) for cediranib and 68·8% (95% CI 40·5–85·6) for placebo (figure 4). Excluding participants with previous cediranib treatment, the log rank p value (one sided) was 0·42.

The safety population included 47 (98%) participants who had at least one dose of trial treatment. The side-effect profile was as expected for a VEGFR inhibitor. The most common adverse events on blinded treatment were diarrhoea, hypertension, fatigue, and nausea (table 2). Most grade 3 adverse events on cediranib were hypertension and diarrhoea, which were managed by dose reduction (adverse events that resulted in doses reductions are not shown separately in table 2). No grade 4 adverse events or deaths occurred on cediranib during the masked phase. 15 serious adverse reactions were reported in 12 participants (appendix p 8). Of these serious adverse reactions, 12 occurred on open-label cediranib. The most common symptoms were dehydration (n=2), vomiting (n=2), and proteinuria (n=2). One (2%) of 48 participants died because of an intracranial haemorrhage 41 days after starting open-label cediranib with no evidence of cerebral metastases. This event was considered as probably related to treatment. All other deaths were unrelated to treatment and due to ASPS.

**Table 2:** Adverse events reported during the blinded treatment phase

	**Cediranib group (n=31)**		**Placebo group (n=16)**	
	Grade 1–2	Grade 3	Grade 1–2	Grade 3
Diarrhoea	26 (84%)	2 (6%)	6 (38%)	0
Hypertension	20 (65%)	6 (19%)	9 (56%)	0
Fatigue	16 (52%)	1 (3%)	6 (38%)	0
Nausea	12 (39%)	0	3 (19%)	0
Dyspnoea	11 (35%)	0	2 (13%)	1 (6%)
Decreased appetite	9 (29%)	0	6 (38%)	0
Arthralgia	9 (29%)	0	2 (13%)	0
Weight decreased	9 (29%)	0	2 (13%)	0
Headache	9 (29%)	0	4 (25%)	0
Hypothyroidism	9 (29%)	0	1 (6%)	0
Cough	8 (26%)	1 (3%)	6 (38%)	0
Abdominal pain	9 (29%)	0	4 (25%)	0
Pain in extremity	7 (23%)	1 (3%)	6 (38%)	0
Constipation	8 (26%)	0	3 (19%)	0
Mucosal inflammation	6 (19%)	1 (3%)	1 (6%)	0
Palmar-plantar erythrodysaesthesia	7 (23%)	0	0	0
Stomatitis	5 (16%)	0	1 (6%)	0
Back pain	5 (16%)	0	1 (6%)	0
Blood bilirubin increased	4 (13%)	0	1 (6%)	0
Dry skin	4 (13%)	0	0	0
Insomnia	4 (13%)	0	2 (13%)	0
Lymphocyte count decreased	3 (10%)	1 (3%)	1 (6%)	0
Upper respiratory tract infection	4 (13%)	0	1 (6%)	0
Asthenia	3 (10%)	1 (3%)	0	0
Neutrophil count decreased	4 (13%)	0	1 (6%)	0
Vomiting	4 (13%)	0	1 (6%)	0
Rash	4 (13%)	0	1 (6%)	0
Nasopharyngitis	3 (10%)	0	4 (25%)	0
Chest pain	4 (13%)	1 (3%)	4 (25%)	0
Proteinuria	3 (10%)	0	2 (13%)	0
Dysphonia	3 (10%)	0	2 (13%)	0
γ-glutamyltransferase increased	3 (10%)	0	0	1 (6%)
Oedema peripheral	2 (6%)	0	2 (13%)	0
Epistaxis	2 (6%)	0	2 (13%)	0
Dry mouth	2 (6%)	0	2 (13%)	0
Haemoptysis	2 (6%)	0	2 (13%)	0
Oropharyngeal pain	2 (6%)	0	2 (13%)	0
Blood amylase increased	0	1 (3%)	2 (13%)	0
Pain	1 (3%)	0	2 (13%)	0
Anaemia	1 (3%)	0	3 (19%)	0
Injection site haematoma	0	0	2 (13%)	0
Pruritus	0	0	2 (13%)	0
Monoparesis	0	0	2 (13%)	0
Lower respiratory tract infection	0	0	2 (13%)	0
Blood alkaline phosphatase increased	1 (3%)	1 (3%)	0	0
Alanine aminotransferase increased	1 (3%)	1 (3%)	0	0
Pyrexia	2 (6%)	1 (3%)	1 (6%)	0
Hypophosphataemia	2 (6%)	1 (3%)	0	0
Amenorrhoea	1 (3%)	1 (3%)	0	0
Partial seizures	0	1 (3%)	0	0

Adverse events were graded according to Common Terminology Criteria for Adverse Events version 4. Adverse events occuring in at least 10% of patients (or one or more patients for grade 3 or worse events) are reported here. There were no grade 4 adverse events or deaths due to these causes.

An unplanned exploratory analysis to determine the effect of previous tyrosine-kinase inhibitors on progression-free survival was difficult to assess because small numbers precluded formal comparisons (appendix p 7).

## Discussion

CASPS has confirmed the activity of cediranib in participants with advanced, metastatic ASPS, with a significant difference in the median percentage change in sum of the diameters of target marker lesions at 24 weeks compared with placebo. The primary endpoint was chosen to show the degree of tumour shrinkage occurring in response to cediranib without being confounded by treatment crossover. This endpoint was considered a more reliable index of treatment effect than progression-free survival in light of known indolent progression, spontaneous stabilisation, and spontaneous regression in this disease.^12^ Additionally, this unconventional endpoint was considered more sensitive than progression-free survival and thus required fewer participants to show a significant difference between the two groups—an important consideration for a trial in such a rare disease. The difference in median progression-free survival at the time of the analysis was not significant, with absolute values of 10·1 months (IQR 5·3–19·0) for cediranib and 4·9 months (1·9–20·0) for placebo. Our findings here suggest that cediranib stabilises metastatic disease and produces objective responses, with 19% of participants given cediranib having a partial response in the first 24 weeks. Some participants with stable disease and partial response had long-lasting disease control, with a median duration of response of 16 months (IQR 15·7–26·0).

We recognise that our analyses of secondary endpoints such as progression-free survival and overall survival are underpowered. However, the rarity and indolence of ASPS and the incorporation of crossover within the randomised trial dictated the use of an unconventional primary endpoint and acceptance that demonstration of superiority using more conventional criteria would not be possible. Progression-free survival and overall survival data are reported to enable comparisons with other drugs, none of which have yet been studied in a prospective randomised trial (eg, sunitinib, pazopanib, axitinib, axitinib plus pembrolizumab, anlotinib). Most data on other tyrosine-kinase inhibitors are derived from reports on small single-centre studies or larger studies in soft-tissue sarcomas of which ASPS comprised only a small component. Toxicity was as expected for a drug of this class and was generally manageable with dose interruptions or reductions, with few participants allocated to cediranib withdrawing due to treatment side-effects. Four participants withdrew subsequently because of patient choice, two because of adverse events, and one died. One death due to a vascular event was deemed probably treatment related because thromboembolic events are a known side-effect of VEGF receptor inhibitors.

The idea that inhibitors of angiogenesis, such as VEGF receptor inhibitors like cediranib, might be active against ASPS was prompted by the observations of dormancy and spontaneous stabilisation, and the belief that some form of angiogenic switch might be responsible.^18^ An additional explanation, which is not mutually exclusive, is that this behaviour could be due to immune surveillance, for which supportive clinical data now exist from the use of immunomodulatory drugs (eg, pembrolizumab).^16^

Evidence of lactate as an energy source in ASPS,^6^ with consequent upregulation of HIF1α and VEGF, might partially explain the activity of drugs that inhibit VEGF receptors, although other targets could be involved. Spontaneous regression could be mediated via the immune system. A study of the VEGF receptor inhibitor axitinib in combination with pembrolizumab investigating immunomodulation in soft-tissue sarcomas (NCT02301039) is ongoing.^16^ In participants with ASPS, the proportion with an objective response was 45·5% (95% CI 18·1–75·4) and with a 3-month progression-free survival estimate of 90·9% (95% CI 50·8–98·7).

To our knowledge, none of the molecularly targeted drugs discussed here have been studied as extensively as cediranib in ASPS or have shown superior activity. A randomised phase 2 study comparing cediranib with sunitinib monotherapy (NCT01391962) is currently recruiting participants and has yet to report any results. This study does not have a no-treatment group, precluding control for spontaneous stabilisation or regression or variations in the rate of disease progression between the groups.

In CASPS, the proportion of participants who had an objective response at week 24 (19%) was lower than that reported overall by Kummar and colleagues in a phase 2 trial of cediranib in metastatic ASPS (35%),^11^ in which participants had a slightly lower median age than in this study (27 years *vs* 31 years), but a similar proportion had received antiangiogenic therapy previously (26% *vs* 33% in CASPS). A key difference between the studies is that participants were not required to have had disease progression in the previous 6 months in Kummar and colleagues' study. Although CASPS was open to participants aged 16 years and older (in Kummar and colleagues' study, the threshold was ≥18 years) only one patient younger than 20 years was recruited, showing the predominance of participating centres only seeing adult patients.

Undertaking a randomised trial in such a rare disease, which has an incidence of less than one per million people per year, was challenging. The decision to undertake CASPS as a randomised trial was vindicated by the fact that seven (44%) of 16 participants in the placebo group had stable disease at 24 weeks, despite all participants having documented progressive disease before study entry. At the time of primary analysis with a minimum of 6 months follow-up after random assignment to treatment, four (25%) participants in the placebo group had still not progressed. Considering these data, following up participants with ASPS for at least 3 months to confirm progressive disease before considering systemic therapy seems reasonable. The decision to allow crossover to cediranib was made on ethical grounds to enable all participants in the study access to the drug at some stage. This choice inevitably led to confounding in the interpretation of overall survival and long-term progression-free survival, as shown by the apparent convergence of the progression-free survival curves at 12 months.

The findings of this study support the concept that antiangiogenic therapy is active against advanced ASPS. Whether or not the spectrum of receptor tyrosine kinases inhibited by cediranib is substantially different from that of other drugs that are active in this disease (eg, sunitinib) is unclear. Based solely on hypertension incidence as a surrogate marker in this and other studies, cediranib appears to be a potent VEGF receptor inhibitor. Comparative data on relative potency in vitro against different receptor tyrosine kinases do not seem helpful in predicting toxicity profiles, and hypertension is not a reliable efficacy biomarker.^19^

Given the high incidence of metastatic disease and poor long-term prognosis of ASPS, together with the lack of efficacy of conventional chemotherapy, the confirmation of significant clinical activity with cediranib in this disease is an important step towards the goal of long-term disease control for these young patients. Further studies using cediranib in ASPS and other sarcomas, in conjunction with other drugs with potential activity in the disease, are warranted.

## Data sharing

The Institute of Cancer Research, Clinical Trials and Statistics Unit (ICR-CTSU) supports the wider dissemination of information from the research it conducts and increased cooperation between investigators. Trial data is collected, managed, stored, shared, and archived according to ICR-CTSU Standard Operating Procedures to ensure the enduring quality, integrity, and utility of the data. Formal requests for data sharing are considered in line with ICR-CTSU procedures with due regard given to funder and sponsor guidelines. Requests are via a standard proforma describing the nature of the proposed research and extent of data requirements. Data recipients are required to enter a formal data sharing agreement that describes the conditions for release and requirements for data transfer, storage, archiving, publication, and Intellectual Property. Requests are reviewed by the Trial Management Group (TMG) in terms of scientific merit and ethical considerations including patient consent. Data sharing is undertaken if proposed projects have a sound scientific or patient benefit rationale as agreed by the TMG and approved by the Independent Data Monitoring and Steering Committee as required. Restrictions relating to patient confidentiality and consent will be limited by aggregating and anonymising identifiable patient data. Additionally, all indirect identifiers that could lead to deductive disclosures will be removed in line with Cancer Research UK Data Sharing Guidelines.
