# Supplementary material for: Cediranib in patients with alveolar soft-part sarcoma (CASPS): a double-blind, placebo-controlled, randomised, phase 2 trial
Source: Lancet Oncol. 2019 Jul;20(7):1023–34. doi: 10.1016/S1470-2045(19)30215-3 (PMC6602919; doi:10.1016/S1470-2045(19)30215-3)
Supplement: Supplementary appendix [file mmc1.pdf]

THE LANCET
Oncology

Supplementary appendix

This appendix formed part of the original submission and has been peer reviewed. We post it as supplied by the authors.

Supplement to: Judson I, Morden J P, Kilburn L, et al. Cediranib in patients with alveolar soft-part sarcoma (CASPS): a double-blind, placebo-controlled, randomised, phase 2 trial. *Lancet Oncol* 2019; published online May 31. http://dx.doi.org/10.1016/S1470-2045(19)30215-3.

Cediranib in alveolar soft part sarcoma (CASPS), a double blind, placebo-controlled randomised phase II trial

Supplementary appendix

CASPS trial participating sites

Site	Principal Investigator	Total recruited
The Royal Marsden NHS Foundation Trust, London, UK	Dr Charlotte Benson	11
The Christie NHS Foundation Trust, Manchester, UK	Dr Michael Leahy	7
University College London Hospitals NHS Foundation Trust, London, UK	Dr Beatrice Seddon	4
Royal Victoria Infirmary and Freeman Hospital, Newcastle-upon-Tyne University Hospitals NHS Trust, Newcastle-upon-Tyne, UK	Dr Quentin Campbell-Hewson	4
Chris O'Brien Lifehouse, Sydney, Australia	Associate Professor Catriona McNeil	4
Princess Alexandra Hospital, Brisbane, Australia	Dr Warren Joubert	4
Nottingham University Hospitals NHS Trust, Nottingham, UK	Dr Ivo Hennig	3
Hospital de la Santa Creu i Sant Pau, Barcelona, Spain	Dr Antonio Lopez-Pousa	3
Hospital Puerta de Hierro, Madrid, Spain	Dr Ricardo Cubedo	3
Hospital Miguel Servet, Zaragoza, Spain	Dr Javier Martinez Trufero	3
Bristol Haematology and Oncology Centre, University Hospitals Bristol NHS Foundation Trust, Bristol, UK	Dr Adam Dangoor	2

Supplementary Figures and Tables

Figure A1: Best percentage change in sum of target marker lesions during the blinded treatment phase

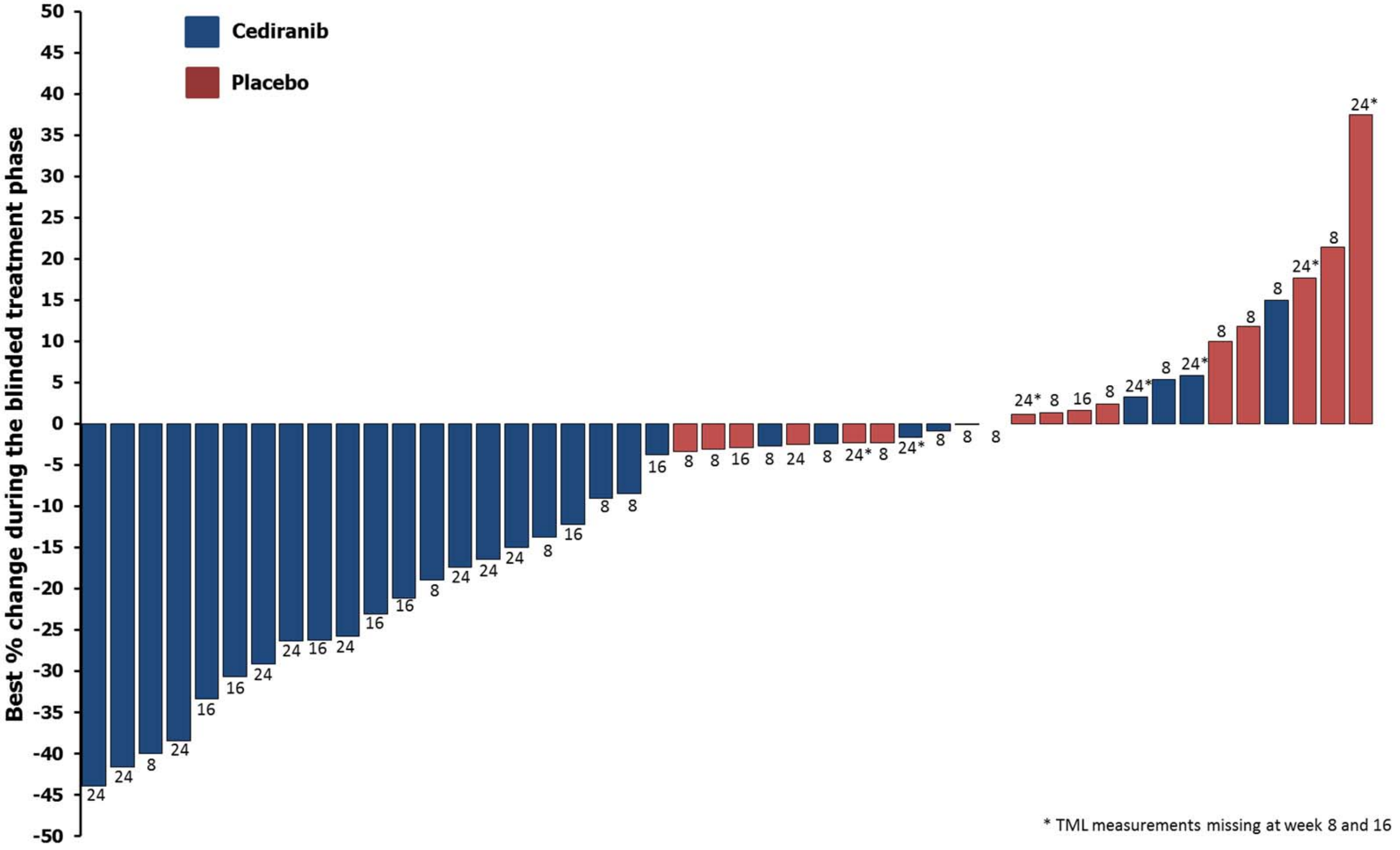

The week at which best response occurred is indicated on the graph for each patient

Figure A2: Spaghetti plot of percentage change in target marker lesions (TMLsum) from baseline in patients randomised to cediranib

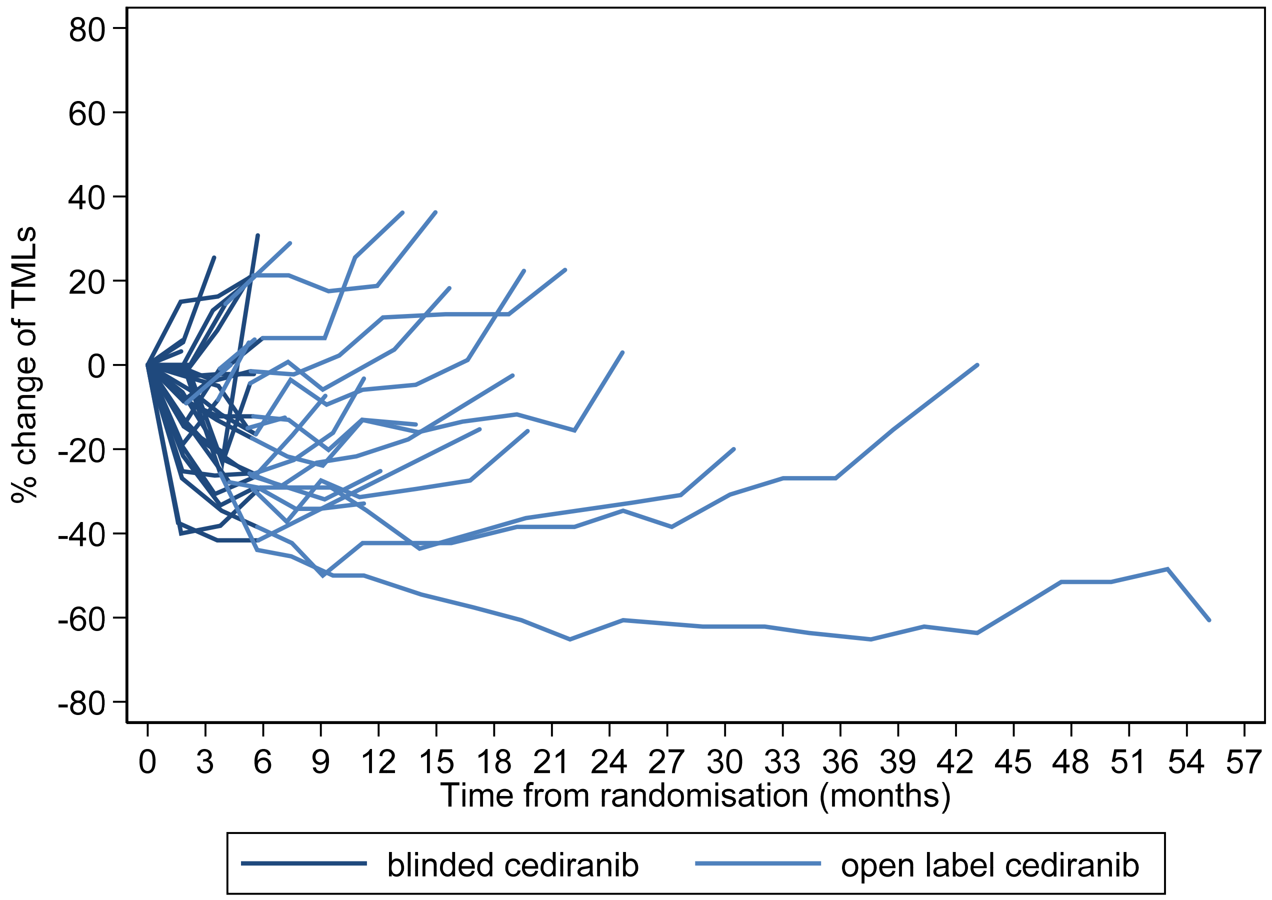

Figure A3: Spaghetti plot of percentage change in target marker lesions (TMLsum) from baseline in patients randomised to placebo

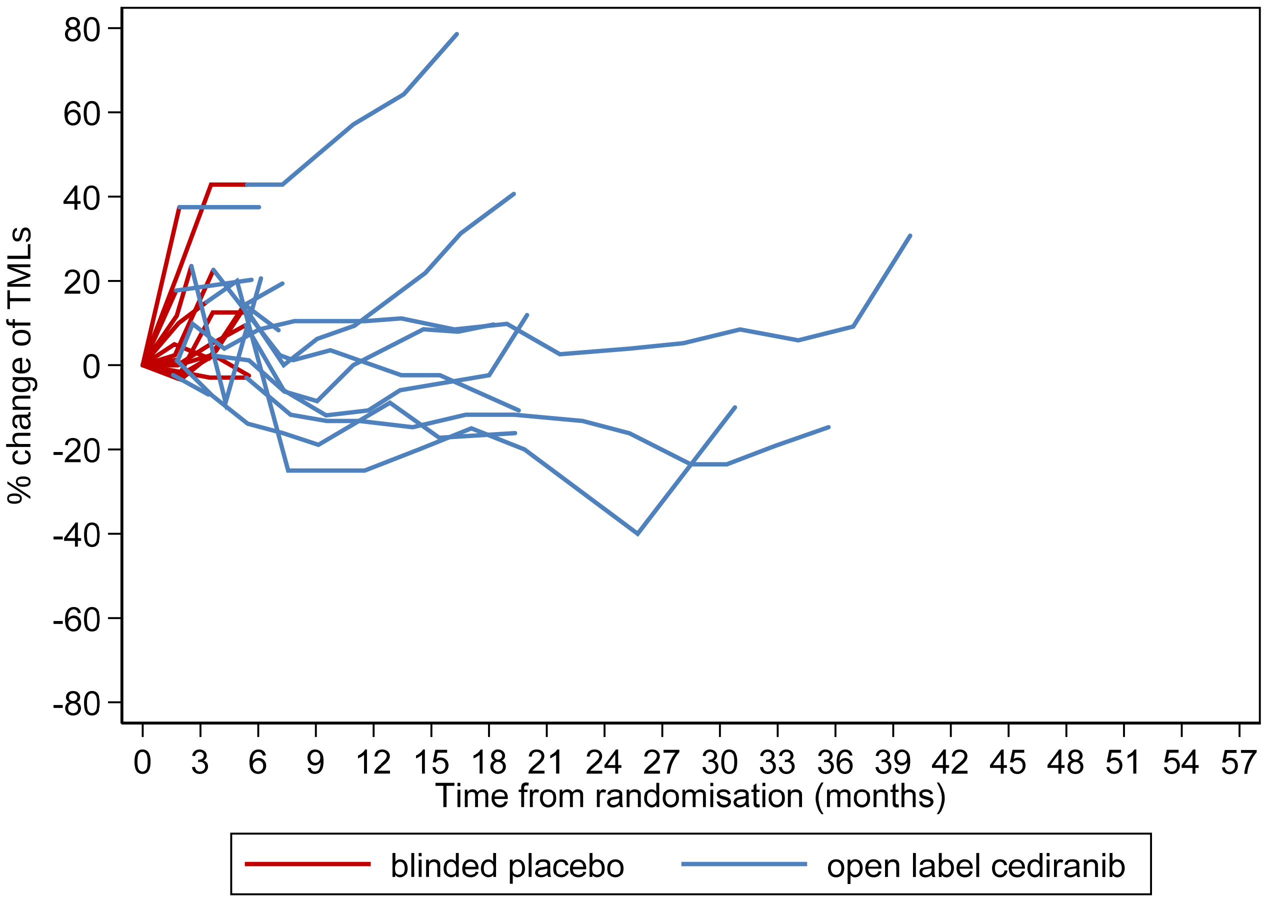

Figure A4: Kaplan Meier curve for progression free survival by treatment group (sensitivity analysis including non-RECIST confirmed progression as an event)

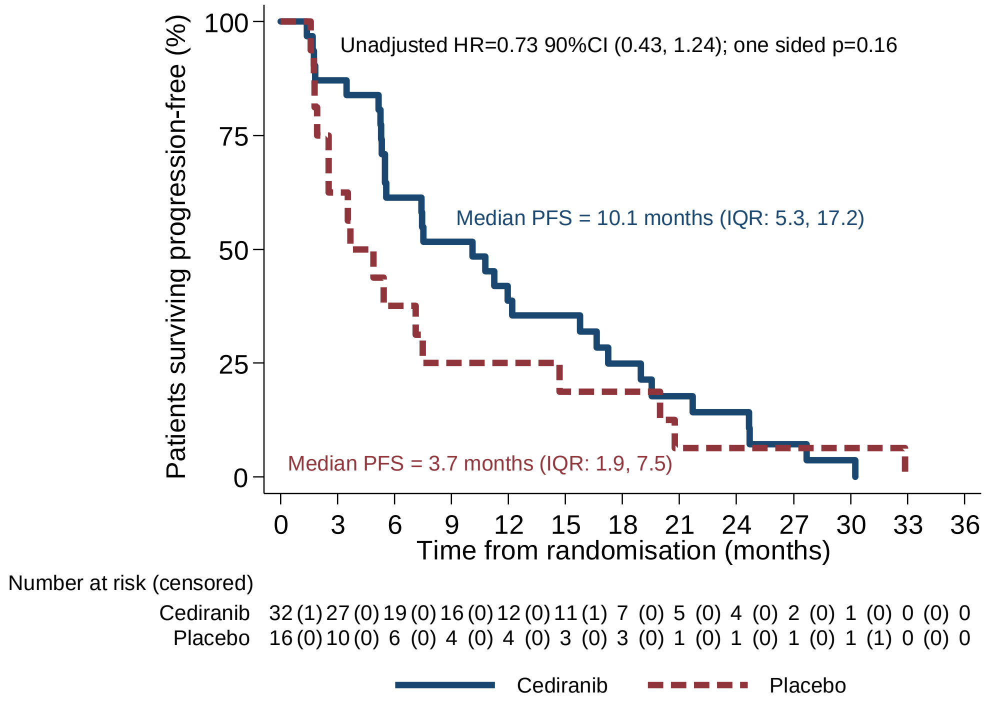

Excluding patients with prior cediranib treatment, unadjusted HR=0.66 (0.39, 1.14); one sided p-value = 0.11

Figure A5: Kaplan Meier curve for progression free survival by treatment group (sensitivity analysis including non-RECIST confirmed progression as an event, censoring patients at 26 weeks after randomisation)

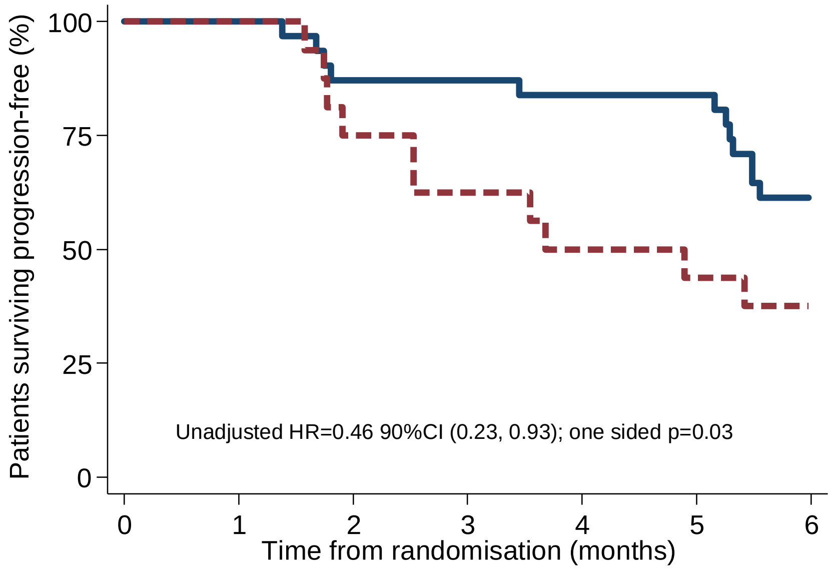

Number at risk (censored)

Cediranib	32	(1)	31	(0)	27	(0)	27	(0)	26	(0)	26	(19)	0
Placebo	16	(0)	16	(0)	12	(0)	10	(0)	8	(0)	7	(6)	0

Cediranib — Placebo

Figure A6: Kaplan Meier curves for progression free survival by previous TKI use in patients randomised to cediranib

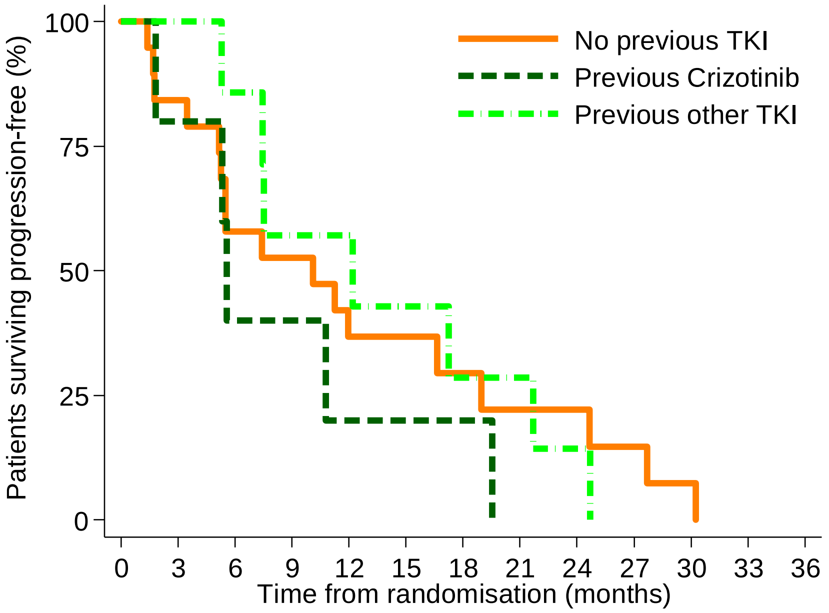

Number at risk (censored)

	0		3		6		9		12		15		18		21		24		27		30		33		36
No previous TKI:	20	(1)	16	(0)	11	(0)	10	(0)	7	(0)	7	(2)	4	(0)	3	(0)	3	(0)	2	(0)	1	(0)	0	(0)	0
Previous Crizotinib:	5	(0)	4	(0)	2	(0)	2	(0)	1	(0)	1	(0)	1	(0)	0	(0)	0	(0)	0	(0)	0	(0)	0	(0)	0
Previous other TKI:	7	(0)	7	(0)	6	(0)	4	(0)	4	(0)	3	(0)	2	(0)	2	(0)	1	(0)	0	(0)	0	(0)	0	(0)	0

Table A1: Serious Adverse Reactions reported in the trial

Patient number	Type	Category	Randomised treatment	Treatment received at time of event [time on that treatment prior to event (weeks)]	Severity	Summary of event (CTC grade)	Outcome
1	SAR	Hospitalisation	Cediranib	Cediranib (open-label) [43.9]	Severe	Dehydration (3); Diarrhoea (3); Vasovagal reaction (3)	Recovered
2	SAR	Hospitalisation	Placebo	Cediranib (open-label) [40.1]	Moderate	Rectal ulcer (2)	Recovered
3	SAR	Hospitalisation	Placebo	Cediranib (open-label) [1.9]	Severe	Tumour pain (right leg) (3)	Recovered
3	SAR	Hospitalisation	Placebo	Cediranib (open-label) [9.1]	Moderate	Swollen right leg, possible DVT (2)	Recovered
4	SAR	Hospitalisation	Placebo	Cediranib (open-label) [131]	Severe	Myocardial infarction (3)	Recovered
5	SAR	Hospitalisation	Cediranib	Cediranib (open-label) [50.7]	Moderate	Superficial infection of surgical wound (3)	Recovered
6	SAR	Hospitalisation	Placebo	Cediranib (open-label) [7.3]	Moderate	Nausea (3); Dehydration (2); Dyspepsia (2); Vomiting (3)	Recovered
6	SAR	Hospitalisation	Placebo	Cediranib (open-label) [10.6]	Moderate	Vomiting (3)	Recovered
6	SAR	Hospitalisation	Placebo	Cediranib (open-label) [15.3]	Severe	Pleuritic chest pain (3); Constipation (2)	Recovered
7	SAR	Other*	Cediranib	Cediranib (blinded phase) [2.1]	Moderate	Arthralgia (2); Oedema limbs (2); Paraesthesia (both feet and right hand) (1)	Condition present at time of patient death
8	SAR	Hospitalisation	Placebo	Cediranib (open-label) [1.9]	Severe	Pharyngitis (2); Urinary tract infection (2); Fatigue (3); Headache (3)	Recovered with sequelae
9	SAR	Death	Placebo	Cediranib (open-label) [5.9]	Fatal	Intracranial haemorrhage (5)	Death
10	SAR	Hospitalisation	Cediranib	Cediranib (open-label) [59.9]	Severe	Proteinuria (3); Thrombotic microangiopathy (3)	Recovered with sequelae
11	SAR	Hospitalisation	Cediranib	Cediranib (blinded phase) [12.9]	Moderate	Proteinuria (2)	Recovered
12	SUSAR	Hospitalisation	Cediranib	Cediranib (blinded phase) [17.4]	Mild	Haematemesis (1)	Recovered

*Clinician felt this was an important medical event that warranted further reporting

CASPS

A Phase II Trial of Cediranib in the Treatment of Patients with Alveolar Soft Part Sarcoma (CASPS)

**Chief Investigator:**	Professor Ian Judson
**Co - Sponsors:**	The Institute of Cancer Research / The Royal Marsden NHS Foundation Trust
**International Peer Review:**	Clinical Trials Advisory & Awards Committee (CTAAC)
**Funders:**	Cancer Research UK<br>AstraZeneca
**Coordinating Trials Unit:**	ICR Clinical Trials and Statistics Unit (ICR-CTSU)<br>The Institute of Cancer Research

PROTOCOL Version 8
Dated 12 October 2018

**EudraCT Number:**	2010-021163-33
**ICR-CTSU Protocol Number:**	ICR-CTSU/2010/10027
**Main REC Reference Number:**	10/H0806/118
**ISRCTN:**	ISRCTN63733470
**Cancer Research UK Reference Number:**	CRUK/10/021
**AstraZeneca Reference Number:**	ISSRECE0036
**IRAS Project ID:**	60384

The CASPS trial has been scientifically approved
by Cancer Research UK's Clinical Trials Advisory & Awards Committee (CTAAC)
The CASPS trial is part of the National Institute for
Health Research Clinical Research Network Trial Portfolio in the UK

ICR The Institute of Cancer Research   CANCER RESEARCH UK

This protocol is a controlled document and should not be copied, distributed or reproduced without the written permission of the ICR-CTSU

Scientific Coordination

Professor Ian Judson (Chief Investigator)
Cancer Research UK Centre for Cancer Therapeutics
Sycamore House
Royal Marsden Hospital
Downs Road
Sutton, Surrey SM2 5PT
Telephone: +44 (0)20 8722 4303
Fax: +44 (0)20 8642 7979
E-mail: Ian.Judson@icr.ac.uk

Trial Coordination

ICR Clinical Trials & Statistics Unit (ICR-CTSU)
Division of Clinical Studies
The Institute of Cancer Research
Sir Richard Doll Building
Cotswold Road
Sutton, Surrey SM2 5NG

This study is coordinated by the ICR-CTSU, a UK Clinical Research Collaboration (UKCRC) registered and National Cancer Research Institute (NCRI) Cancer clinical trials unit. Institutions outside of the UK will participate either as individual sites with trial coordination by the ICR-CTSU, or through national Sarcoma Groups. The country-specific trial coordination duties delegated to each Sarcoma Group will be defined in a contract between the Sarcoma Group and the co-sponsors.

ICR-CTSU Scientific Lead:
Judith Bliss
Tel: +44 (0)20 8722 4297
Judith.Bliss@icr.ac.uk

ICR-CTSU Statistician:
Lucy Kilburn
Tel: +44 (0)20 8722 4080
Lucy.Kilburn@icr.ac.uk

Any questions relating to this protocol should be addressed in the first instance to the CASPS Trial Manager within ICR-CTSU:

Email: casps-icrctsu@icr.ac.uk
General enquiries: +44 (0)208 722 4152
Fax: +44 (0)208 770 7876

Protocol Development Group

Judith Bliss	Professor of Clinical Trials	ICR-CTSU, The Institute of Cancer Research, Sutton, Surrey
Ian Judson	Clinical Consultant - Cancer Therapeutics	Cancer Research UK Centre for Cancer Therapeutics, The Institute of Cancer Research & The Royal Marsden Hospital
David Olmos	Senior Clinical Research Fellow	Cancer Research UK Centre for Cancer Therapeutics, The Institute of Cancer Research & The Royal Marsden Hospital
James Morden	Trial Statistician	ICR-CTSU, The Institute of Cancer Research, Sutton, Surrey
Claire Snowdon	Deputy Director (Operations)	ICR-CTSU, The Institute of Cancer Research, Sutton, Surrey
Lisa Fox (previously Rogers)	Senior Trial Manager	ICR-CTSU, The Institute of Cancer Research, Sutton, Surrey

The Trial Management Group (TMG) will be constituted from members of the Protocol Development Group, and Principal Investigators from a subset of participating centres, including international Sarcoma Group leads and country Lead Investigators. A list of current members can be obtained from the CASPS Trial Manager at ICR-CTSU.

Protocol Authorised by:

Name & Role	Signature	Date
Professor Ian Judson (Chief Investigator)	I Judson	12/10/2018

This protocol describes the CASPS trial and provides information about procedures for entering patients into this trial. The protocol should not be used as a guide for the treatment of other patients.

Every care was taken in the preparation of this protocol, but corrections or amendments may be necessary. These will be circulated to investigators in the trial, but centres entering patients for the first time are advised to contact ICR-CTSU to confirm they have the most recent version. Protocol amendments will be circulated to participating centres as they occur.

This clinical trial will be conducted in compliance with the protocol, all international guidelines, national laws and regulations of the countries in which the Clinical Trial is performed, as well as any applicable guidelines.

CONTENTS

1. TRIAL SUMMARY 6
2. INTRODUCTION 7
2.1. Alveolar Soft Part Sarcoma 7
2.2. Cediranib 7
2.2.1. Pre-clinical experience with cediranib 8
2.2.2. Clinical experience with cediranib 8
2.2.3. Pharmacokinetic profile 9
2.2.4. Pharmacokinetic profile and tumour response 10
2.2.5. Safety profile 11
2.2.6. Experience of cediranib in ASPS to date 13
2.3. Rationale for Study 14
3. TRIAL OBJECTIVES 14
3.1. Primary Objective 14
3.2. Secondary Objectives 14
3.3. Exploratory Objectives 15
4. TRIAL DESIGN 15
5. STUDY FLOW CHART 17
6. PATIENT SELECTION & ELIGIBILITY 18
6.1. Number of Patients 18
6.2. Source of Patients 18
6.3. Inclusion Criteria 18
6.4. Exclusion Criteria 18
7. CONSENT 20
7.1. Procedure for Obtaining Informed Consent 20
8. SCREENING 20
8.1. Clinical Evaluations 20
8.2. Pathology Review 21
8.3. Optional Research Samples (see Laboratory Manual for methods) 21
9. RANDOMISATION 22
9.1. Randomisation Procedure 22
10. SCHEDULE OF ASSESSMENTS 23
11. TRIAL ASSESSMENTS 25
11.1. Baseline (and / or Cycle 1 Day 1) (Where 1 cycle is 28 days) 25
11.2. On-treatment Assessments 25
11.2.1. Week 1 (Cycle 1 Day 8, +/- 2 days) 25
11.2.2. Week 2 (Cycle 1 Day 15, +/-1 day) 25
11.2.3. Week 3 (Cycle 1 Day 22, +/- 2 days) 25
11.2.4. Week 4 (Cycle 2 Day 1, +/- 2 days) 26
11.2.5. Weeks 4-24, every 4 weeks (+/- 3 days); Weeks 24-48, every 8 weeks (+/- 3 days); Week 48 onwards, every 12 weeks (+/- 1 week) 26
11.2.6. Weeks 8 – 48, every 8 weeks (+/- 3 days); Week 48 onwards, every 12 weeks (+/- 1 week) 26
11.2.7. Weeks 8 – 52, every 8 weeks (+/- 3 days); Week 48 onwards, every 12 weeks (+/- 1 week) 26
11.3. Treatment Discontinuation (for all patients at the time of discontinuation of treatment) 27
11.4. Optional Research Sample Collection (see Laboratory Manual for detail and methods) 27
12. UNBLINDING 28
12.1. Procedure for Systematic Unblinding of all Patients 28
12.2. Emergency unblinding 28
13. TREATMENT DURATION AND DISEASE PROGRESSION 29
14. SAFETY FOLLOW UP (30 DAYS AFTER TREATMENT DISCONTINUATION) 29
15. FOLLOW UP 30
16. TRIAL TREATMENT 30
16.1. Drug Dose 30

16.2. Drug Schedule....30
16.3. Drug Manufacturer....31
16.4. Drug Presentation, Packaging and Labelling....31
16.5. Drug Distribution....31
16.6. Drug Storage....32
16.7. Drug Compliance and Accountability....32
16.8. Emergency Code Breaking....32
16.9. Duration of Trial Treatment....33
17. TOXICITY MANAGEMENT....33
17.1. Dose Delays/Modifications....33
17.2. Hypertension....34
17.3. Posterior reversible encephalopathy syndrome....34
17.4. Diarrhoea....35
17.5. Fatigue....36
17.6. Hand and Foot syndrome....36
17.7. Proteinuria....36
17.8. Thrombocytopenia....36
17.9. Abnormal Liver Function....37
17.10. Haemorrhage....37
17.11. Thyroid dysfunction: hypothyroidism and hyperthyroidism....37
17.12. Renal Function....38
18. TREATMENT PRECAUTIONS....38
18.1. Surgery during Cediranib Treatment....38
18.2. Pregnancy and Breast Feeding....39
18.3. Blood Donors....39
18.4. Bone Growth....39
18.5. Concomitant Medications....39
19. PHARMACOVIGILANCE....40
19.1. Adverse Event Definitions....40
19.2. Causality (Relationship to Study Drug)....41
19.3. Expectedness....42
19.4. Reporting Serious Adverse Events (SAEs) to ICR-CTSU....42
19.5. Pregnancy....42
19.6. Review of Serious Adverse Events (SAEs)....43
19.7. Expedited Reporting of SUSARs....43
19.8. Unblinding of SUSARs....44
19.9. Follow up of Serious Adverse Events....44
19.10. Annual Reporting of Serious Adverse Reactions (including SUSARs)....44
19.11. Flow diagram for SAE reporting, and action following report....46
20. STATISTICAL CONSIDERATIONS....47
20.1. Trial Design....47
20.2. Randomisation....47
20.3. Primary Endpoint....47
20.4. Secondary Endpoints....47
20.5. Exploratory Endpoints....47
20.6. Statistical Background....47
20.7. Statistical Design....48
20.8. Interim Analysis....48
20.9. Analysis Plan....48
20.10. Endpoint Definitions....49
20.11. Independent Data Monitoring Committee....50
21. TRIAL MANAGEMENT....50
21.1. Trial Management Group (TMG)....50
21.2. Joint Independent Data Monitoring Committee and Trial Steering Committee....50
22. RESEARCH GOVERNANCE....51
22.1. Co-sponsor Responsibilities....51
22.2. Sarcoma Group Responsibilities....51
22.3. Principal Investigators Responsibilities....51
22.4. AstraZeneca Responsibilities....52

23. TRIAL ADMINISTRATION & LOGISTICS 53
23.1. Data Acquisition 53
23.2. Central Data Monitoring 53
23.3. On-Site Monitoring 53
23.4. Completion of the Study and Definition of Study End Date 54
23.5. Archiving 54
24. PATIENT PROTECTION 54
24.1. Patient Confidentiality 54
24.2. Data Protection 55
24.3. Liability and Insurance 55
25. FINANCIAL MATTERS 55
26. PUBLICATION POLICY 56
REFERENCES 57
APPENDIX 1: RECIST CRITERIA GUIDELINES 58
APPENDIX 2: ECOG PERFORMANCE STATUS 63
APPENDIX 3: HYPERTENSION MANAGEMENT GUIDELINES 64

1. TRIAL SUMMARY

**PROTOCOL TITLE**	A Phase II Trial of Cediranib in the Treatment of Patients with Alveolar Soft Part Sarcoma (CASPS).
**TARGET DISEASE**	Alveolar soft part sarcoma (ASPS).
**STUDY OBJECTIVES**	To confirm the ability of cediranib to halt disease progression in patients with metastatic ASPS, as measured by the change in tumour size at 24 weeks after randomisation, and to produce objective response according to RECIST criteria.
**STUDY DESIGN**	Two-arm, randomised, double-blind, international, multi-centre phase II trial.
**TRIAL POPULATION**	Patients aged 16 years and older with a histologically confirmed diagnosis of ASPS.
**TREATMENT REGIMEN**	Patients will be randomised to receive cediranib (30 mg daily po) or placebo (30 mg daily po) in a 2:1 ratio. At 24 weeks post randomisation, treatment will be unblinded after which time all patients on placebo and those who have not progressed on active treatment will be given cediranib. Treatment will then continue until objective disease progression or death.
**RECRUITMENT TARGET**	36 patients.
**PRIMARY ENDPOINT**	Week 24 (or progression if sooner) percentage change in the sum of target marker lesions from randomisation.
**SECONDARY ENDPOINTS**	Week 24 response rate (RECIST).<br>Best response (RECIST) and best reduction (%) in tumour size.<br>Progression-free survival.<br>Percentage alive and progression-free at 12 months (APF12).<br>Overall survival.<br>Safety and tolerability profile of cediranib in patients with ASPS.
**EXPLORATORY ENDPOINTS**	Utility and applicability of Choi response in ASPS patients treated with cediranib.<br>Tissue markers of tumour response from archived biopsies and optional pre- and post- treatment biopsies.<br>Evaluation of circulating markers of angiogenesis from blood samples of cediranib treated patients.<br>Evaluation of circulating endothelial/precursor cells in cediranib treated patients.
**FOLLOW UP**	Patients will be assessed for progression every 8 weeks for the first 12 months, and every 12 weeks thereafter. All patients will be followed up until disease progression has been confirmed and for 30 days after last dose of study medication (or death if sooner), or withdrawal of consent for further follow up.

2. INTRODUCTION

2.1. Alveolar Soft Part Sarcoma

Alveolar soft part sarcoma (ASPS) is a rare histological subtype of soft tissue sarcoma. It is morphologically distinct, not associated with any known cell type and consists of nests of cells separated by fibro vascular septae and vascular channels, with a pseudo alveolar pattern, hence the name. It occurs in younger patients, with a slight female predominance and, although usually low grade and indolent, is associated with a high incidence of pulmonary and brain metastases[1]. It is characterised by an unbalanced translocation t(X;17)(p11:p25) which results in the fusion of the ASPL (also known as ASPSCR1) and TFE3 genes. This appears to place the transcription factor TFE3 under the control of the ASPL promoter resulting in unregulated transcription of TFE3 controlled genes.

In spite of the high metastatic rate, the short and medium term prognosis is reasonable, with median survival from the time of diagnosis of metastatic disease reported as >36 months[2], whereas for the majority of soft tissue sarcomas this would be 12 months. However, conventional chemotherapy has no role in the management of this disease [2,3] and thus therapeutic options for these patients remain limited beyond the palliative indication of radiotherapy and/or surgery for symptomatic control.

One of the intriguing clinical features of ASPS is that metastases can appear to become dormant, in that they may reach a certain size and then become stable over long periods. It has been demonstrated that a variety of angiogenesis promoting genes are upregulated in ASPS [4], raising the possibility that dormancy is governed by angiogenesis.

2.2. Cediranib

Cediranib is a potent inhibitor of all 3 VEGF receptors (VEGFR-1, -2 and -3) at nanomolar concentrations. Inhibition of VEGF signalling leads to the inhibition of angiogenesis, lymphangiogenesis, neovascular survival, and vascular permeability. Cediranib also inhibits c-Kit tyrosine kinase. Cediranib inhibited the growth of tumours in preclinical models in a dose-dependent manner. A reduction in microvessel density and metastasis was also observed in these preclinical models. Collectively, these changes indicate that cediranib inhibits tumour growth, metastases and vascular permeability. Consequently, cediranib is currently in clinical development to investigate its effectiveness as a once-daily oral treatment for cancer.

Several cediranib randomised phase II studies are ongoing, including the NCIC's BR29, in 1st line NSCLC and ICON6, a 2nd line Ovarian Cancer study.

2.2.1. Pre-clinical experience with cediranib

For full details of the pre-clinical information, please refer to the Investigator Brochure.

2.2.2. Clinical experience with cediranib

At the time of writing this protocol, approximately 4200 patients have received cediranib.

Cediranib is being evaluated in a broad clinical development programme that encompasses a range of tumour types, and includes both monotherapy and combination studies. For details of the design, status and location of clinical studies with cediranib, please refer to the Investigator Brochure. Patients are also being enrolled into collaborative studies sponsored by the US National Cancer Institute (NCI), the Medical Research Council (MRC), and the National Cancer Institute of Canada Clinical Trials Group (NCIC CTG). These studies include a range of different tumour types. There are no clinical studies with cediranib involving healthy subjects.

Cediranib is being investigated for activity in treating non-small cell lung cancer (NSCLC). The NCIC CTG conducted a randomised, double blind, placebo-controlled Phase II/III study of the efficacy of cediranib 30 mg in combination with standard carboplatin/paclitaxel chemotherapy (BR24). Overall, the tolerability profile for cediranib 30 mg was broadly consistent with previous cediranib studies but there was an imbalance in the number of reports of serious adverse events with a fatal outcome between the patients on the cediranib arm compared with those in the placebo arm which led to study closure and subsequent redesign to the lower dose of cediranib (20 mg).

Cediranib is currently in Phase II development in a number of different tumour types, including ovarian, renal and breast cancer. Recently 2 large phase III studies in colorectal cancer have provided the following data:
HORIZON II (D8480C00051), a randomised, double-blind, phase III study which compared the efficacy and safety of cediranib when added to 5-Fluorouracil, Leucovorin and Oxaliplatin (FOLFOX) or Capecitabine and oaliplatin (XELOX) with placebo plus FOLFOX or XELOX in patients with previously untreated metastatic colorectal cancer. Cediranib met the co-primary endpoint of progression free survival (PFS) but showed no improvement on overall survival (OS) (Confidential communication from AstraZeneca).

HORIZON III (D8480C00013), a randomised, double-blind, multicentre phase II/III study compared the efficacy of cediranib in combination with FOLFOX to Bevacizumab in combination with FOLFOX in patients with previously untreated metastatic colorectal cancer. Cediranib did not meet the primary endpoint superiority or non-inferiority vs

bevacizumab on the primary endpoint of PFS (Confidential communication from AstraZeneca).

No new safety issues were identified that would affect the management of patients in ongoing studies

2.2.3. Pharmacokinetic profile

Following single and multiple oral doses, absorption is extended with C_{max} typically attained between 1 and 8 hours post-dose. Although absolute bioavailability has not been determined, cediranib appears well absorbed with apparently linear PK for single and multiple doses ranging from 0.5 to 60 mg. After attaining C_{max}, plasma concentrations decline in an apparent bi-exponential manner, with a $t½\lambda z$ of 22.0 $\pm$ 6.50 hours.

Dose-proportional increases in $C_{ss,max}$ and AUC_{ss} were observed for cediranib doses ranging from 0.5 to 60 mg. However, further PK data are needed to make a definitive statement about linearity or dose proportionality.

In vitro studies suggested that cytochrome P450 (CYP) enzymes were not significantly involved in the production of the principal human metabolites of cediranib, therefore co-administration of known inhibitors or inducers of hepatic CYP enzymes would not be expected to have significant effects on the clearance of cediranib. However, since potent inhibitors or inducers of CYP enzymes can also affect drug disposition by interaction with transporter proteins and Phase II metabolism, 2 clinical studies have been completed to investigate the PK of cediranib when co-administered with a potent inhibitor (ketoconazole, study D8480C00020) and a potent inducer (rifampicin, study D8480C00029).

Co-administration of ketoconazole 400 mg modestly increased the AUC_{ss} and $C_{ss,max}$ of cediranib 20 mg. Given the relatively small magnitude of these increases and the known variability in the PK of cediranib, the effect was not considered to be clinically significant. Concomitant administration of cediranib with ketoconazole for 3 days did not result in any unexpected safety issues.

Co-administration of rifampicin 600 mg resulted in a decrease in the AUC_{ss} and $C_{ss,max}$ of cediranib 45 mg. Given the relatively low magnitude of these decreases and the known variability in the exposure of cediranib, the effect was considered to be of limited clinical significance. The safety of cediranib was not affected by co-administration of rifampicin on days 8 to 14 of cediranib administration.

Following a single 45 mg dose in the presence and absence of a standard high-fat meal, data showed that food decreases the C_{max} by 33% and AUC by 24%. Cediranib should be administered at least 1 hour before or 2 hours after food. Comparison of studies conducted in Japanese patients, with corresponding Western study data indicates similar PK between Western and Japanese patients.

2.2.4. Pharmacokinetic profile and tumour response

Initial pharmacodynamic assessments from the Phase I clinical study programme have produced indications of potential biological activity in the patient populations studied. At doses of 20 mg and higher, cediranib has demonstrated reductions in blood flow (as detected by Dynamic Contrast Enhanced Magnetic Resonance Imaging [DCE-MRI]) in hepatic metastases in patients with solid tumours and metastatic liver disease.

Phase I data from the cediranib programme showed that cediranib 20 mg was the lowest dose identified with evidence of biological activity (in terms of reductions in tumour blood flow and permeability, blood pressure increases, reductions in sVEGFR-2, and tumour response); however, only a few patients were studied at doses below 20 mg (Drevs et al, 2007). The Phase I data also showed that following multiple doses of cediranib 20 mg, the unbound minimum steady-state concentration in plasma during the dosing interval ($C_{ss,min}$) is 4.86 times above the IC_{50} for inhibition of VEGF-stimulated human umbilical vein endothelial cell proliferation.

Initial biomarker assessments have shown increases in serum VEGF, basic Fibroblast Growth Factor (bFGF) and Placental Growth Factor (PLGF) and reductions in soluble VEGFR-2 (sVEGFR-2) levels. Decreases in sVEGFR-2 levels may be a surrogate for decreased angiogenesis and changes in VEGF could potentially be indicative of acute vascular effects.

VEGF increases have been observed on treatment with cediranib. This was observed across studies in patients treated with cediranib as monotherapy or in combination with other agents, including fulvestrant. Increases in VEGF from baseline were seen as early as 8 h after start of treatment in some studies, where samples were taken at this early time-point. When early time points were evaluated, there was an early peak of VEGF increase, followed by a decrease, though levels remained above baseline during treatment. Decreases in sVEGFR-2 have been seen on cediranib. This was observed across studies in patients treated with cediranib as monotherapy or in combination with other agents, including fulvestrant. Decreases in sVEGFR-2 appeared to be time-dependent, and in some studies, a dose dependency was observed.

2.2.5. Safety profile

To date, approximately 7470 patients with advanced cancer have entered cediranib clinical studies, with an estimated 5550 having received cediranib either as monotherapy or in combination with other anti-cancer agents.

In the first study of cediranib in man, cediranib 45 mg was determined to be the maximum tolerated dose in the monotherapy setting; however, this was based on relatively short-term treatment. Following subsequent evaluation of all available safety and tolerability data from both AstraZeneca-sponsored and collaborative group studies in patients with a range of tumour types, cediranib 30 mg was considered more likely than 45 mg to be the maximum monotherapy dose that would be tolerated with long-term treatment.

Cediranib 20 mg is the recommended starting dose when given in combination with chemotherapy agents, though some exceptions may be appropriate, depending on eg. patient population, tumour type, or the agent to be given in combination with cediranib.

Diarrhoea is one of the most commonly reported adverse reactions, mainly CTCAE Grade 1 to 2. Diarrhoea usually starts early (within the first cycle of treatment), however, it can occur at any time during treatment.

Hypertension is an expected Adverse Event (AE) with agents that inhibit VEGF signalling. In cediranib studies, increases in blood pressure (BP) have been observed and cases of hypertension have been reported, including CTCAE Grade 4 hypertension and end organ damage related to hypertension, such as cerebrovascular events.

Left ventricular dysfunction, in some cases leading to cardiac failure, has been observed in patients receiving cediranib with risk factors for left ventricular dysfunction (including previous or concomitant anthracycline treatment).

A number of events of bleeding and haemorrhage have occurred, they were mostly mild and the most common type of bleeding was mucocutaneous (epistaxis). However, gastrointestinal, central nervous system, pulmonary bleeding and haematuria have also been reported. Some events of haemorrhage were fatal but causality could not be unequivocally assigned to cediranib.

Gastrointestinal perforation and fistula, often reflecting the location of the underling malignancy including vaginal fistula in ovarian cancer patients, have been observed in patients receiving cediranib. Some events of gastrointestinal perforation have been fatal but causality could not be unequivocally assigned to cediranib.

Arterial thromboembolism (including cerebrovascular accident, myocardial infarction and infrequent retinal arterial occlusion) and venous thromboembolic events (including pulmonary embolism and retinal vein occlusion) have been observed in patients receiving cediranib.

Fatigue, nausea, headache, vomiting, anorexia and weight loss are commonly occurring AEs in cediranib studies. Dehydration has been observed in clinical studies as a consequence of cediranib-related or chemotherapy-related diarrhoea, vomiting; anorexia, or reduced oral intake. Hand and foot syndrome was reported in patients treated with cediranib 20 mg in combination with a 5-fluorouracil based chemotherapy and at doses of ≥30 mg in monotherapy and in combination with other chemotherapy.

Hoarseness (dysphonia) has been reported as common and dose-related.

Muscle weakness, pain in extremity, proteinuria, dry mouth, stomatitis, mucosal inflammation and oral pain have been observed in cediranib studies, mainly when the drug is given in combination with chemotherapy, e.g. cisplatin and paclitaxel. Impaired wound healing, generally low grade has been seen. Infections, generally low grade, have been reported, including urinary tract infections in females.

Posterior reversible encephalopathy syndrome (PRES), including reversible posterior leukoencephalopathy syndrome (RPLS), has been reported in patients receiving cediranib in clinical studies.

Increases in transaminases, which are sometimes associated with increases in total bilirubin, have been seen.

Thrombocytopenia and neutropenia, mainly mild and moderate in severity, have been reported with cediranib monotherapy. However, when cediranib is combined with chemotherapy, more frequent and more severe neutropenia and thrombocytopenia have been observed as compared to chemotherapy alone. For patients who received cediranib in combination with platinum and paclitaxel, febrile neutropenia was more frequent as compared to patients receiving chemotherapy alone (both in ovarian and non-small cell cancer), as was neutropenic sepsis in ovarian cancer patients.

Thyroid dysfunction has been observed in patients treated with cediranib, which included dose-related increases from baseline in TSH levels sometimes associated with clinical hypothyroidism, and hyperthyroidism which was mostly mild/moderate and self-limiting.

Pancreatitis has been infrequently reported in patients receiving cediranib.

Dyspnoea, hypomagnesaemia and rash have been reported in patients who received cediranib in combination with a platinum and paclitaxel.

Dysgeusia, oral pain, muco-epithelial dryness, pruritus, arthralgia, back pain, peripheral oedema, ALP increased, blood creatinine increased and hypocalcaemia have been reported in ovarian cancer patients treated with cediranib in combination with a platinum and paclitaxel, followed by cediranib maintenance monotherapy.

An imbalance in incidence of convulsions was observed in patients in a glioblastoma study (with more cases seen on cediranib than placebo/comparator).

2.2.6. Experience of cediranib in ASPS to date

At the time of writing in October 2010, our experience to date with cediranib in ASPS consists of seven patients in 2 separate trials. In phase I clinical trials hypertension was identified as a significant toxicity of cediranib[5]. The first ASPS patient, with newly diagnosed metastatic disease in lungs and axilla, entered a study (AstraZeneca study D8480C00038) investigating the impact of two different doses of cediranib on blood pressure and the benefit or otherwise of prophylactic antihypertensive medication in a 2 x 2 randomisation design. This patient experienced significant tumour shrinkage and remained in remission for nearly two years.

The remaining patients were treated in a study (AstraZeneca study D8480C00046) principally designed to investigate the impact of cediranib on gastrointestinal stromal tumour (GIST) refractory to imatinib and sunitinib but open to a limited number of patients with other sarcomas. The two centres participating in the study, the Royal Marsden Hospital, London and Christie Hospital, Manchester, were able to identify 6 eligible patients

with metastatic ASPS. All of these patients exhibited tumour shrinkage and prolonged disease control and median duration of treatment was >35 weeks. Toxicities were manageable and included fatigue (n=6), diarrhoea (n=5), stomatitis (n=4), headache (n=3) and hypertension (n=3). Five patients had an objective remission according to RECIST and responses were observed in lung, soft tissue and brain.

2.3. Rationale for Study

ASPS is rare, with an incidence in the region of 0.5 – 1% of all sarcomas. Given an approximate annual incidence of sarcomas for the UK of 2,500 (excluding advanced gastrointestinal stromal tumour) a reasonable estimate of incidence for ASPS is 15 per annum. However, within this number the incidence of metastatic disease is high; patients typically survive for well in excess of 3 years with slowly progressive metastatic disease.

There is no standard accepted therapy for this patient group. The only report of clinical benefit concerned a similar agent, sunitinib, also an inhibitor of VEGFR, as well as of other targets[6]. However, in the report of the 5 patients treated only 2 had a partial response and 2 progressed.

The limited evidence available suggests that cediranib has unprecedented activity in ASPS and as such, this needs to be confirmed. Owing to the indolent nature of the disease in many cases, and hence the difficulty in proving that disease stabilisation is due to treatment, a formal prospective comparative study is required. The National Cancer Institute is conducting a phase II study of cediranib in patients with ASPS (NCT00942877) in the USA[7]. However, this study has response rate as its primary endpoint and will not define progression-free survival. The randomised design proposed in CASPS represents the best way of proving that the drug is active in this disease.

3. TRIAL OBJECTIVES

3.1. Primary Objective

The primary objective of this study is to evaluate the efficacy of cediranib in the treatment of ASPS by measuring the percentage change in the sum of target marker lesion diameters from randomisation to week 24 (or progression if sooner) compared to treatment with placebo.

3.2. Secondary Objectives

The secondary objectives of the study are to evaluate:

- Response rate at week 24, best response using RECISTv1.1 and best reduction (%) in tumour size

- Progression-free survival and percentage alive and progression-free at 12 months (APF12)
- Overall survival
- The safety and tolerability profile of cediranib in patients with ASPS

3.3. Exploratory Objectives

- To explore the utility and applicability of Choi response criteria[8] in ASPS patients treated with cediranib.
- To explore tissue markers of tumour response to cediranib in original archived biopsies, and optional pre- and post-treatment biopsies.
- To evaluate the changes in circulating markers of angiogenesis from blood samples in response to cediranib.
- To evaluate the changes in circulating endothelial cells / endothelial precursor cells in response to cediranib.

4. TRIAL DESIGN

This is a randomised phase II, multi-centre, double-blind, placebo-controlled clinical trial. Patients with progressive metastatic histologically confirmed ASPS will be eligible to participate. All patients will be randomised in a 2:1 ratio to treatment with cediranib or matching placebo. Patients and clinicians will be blinded to the study treatment.

Patients will be required to take one 30 mg tablet or two 15 mg tablets of cediranib or matching placebo once a day. At 24 weeks, treatment will be unblinded after which time all patients on placebo and those who have not progressed on active treatment will be given cediranib. Treatment will then continue until objective disease progression, or death if sooner.

Evaluation of tumour response will be according to RECIST v1.1 (Response Evaluation Criteria in Solid Tumours) criteria[9]. The RECIST v1.1 guidelines for measurable, non-measurable, target and non-target lesions and the objective tumour response criteria (complete response, partial response, stable disease or progression of disease) are presented in Appendix 1. All patients will have imaging every 8 weeks during the first 12 months in order to identify time to disease progression with reasonable accuracy. After 12 months the interval between scans will be every 12 weeks. Patients will be followed up until disease progression is confirmed or death.

In addition, the utility and applicability of Choi response criteria in contrast-enhanced images from follow-up CT scans will be evaluated. Patient CT scans from assessments at

the time of screening, Week 24 and the time of disease progression will be saved to CD and sent to the coordinating centre for independent, central assessment of this criteria.

Adverse events, including toxicity to treatment will be assessed according to The National Cancer Institute (NCI) Common Terminology Criteria (CTC) Version 4 (http://ctep.cancer.gov/reporting/ctc.html). Safety and tolerability assessments will be performed every week during the first 4 weeks, and then 4 weekly until the first 24 weeks. Thereafter, safety and tolerability assessments will be performed with the same frequency as radiological disease evaluations.

Patients who are unblinded at the 24 week visit, and found to be taking placebo may start cediranib treatment. These patients will restart all study assessments from Day 1 of Cycle 1 i.e. weekly assessments in the first 4 weeks, 4 weekly visits for the next 24 weeks etc. The schedule of radiological evaluations is the only exception to this, these scans will not restart from Day 1. Patients will continue with the same schedule of scans as previously (8 weekly in the first 12 months from randomisation and 12 weekly thereafter). For all patients (both those starting on active treatment or placebo) duration of treatment and the time period to progression will be calculated from randomisation to treatment, rather than the start of active treatment.

In addition, this study will include a number of exploratory investigations including the evaluation of predictive markers of tumour response to cediranib in original archived biopsies. Separate consent will also be sought for optional pre- and post-treatment biopsies performed within 1 month of treatment starting, after 2 weeks of treatment starting, and if possible at disease progression. Optional peripheral blood samples will be collected for investigations including estimation of circulating markers of angiogenesis and circulating endothelial cells. Details of whether patients have donated tissue samples to a tissue biobank for research purposes prior to trial entry will be requested. If additional samples donated to a tissue biobank are available and the types of sample are believed by the Chief Investigator to be of additional value to the samples already collected within the trial, these samples will be requested from the biobank. Consent to use these samples for the CASPS trial will be obtained from the patient.

5. STUDY FLOW CHART

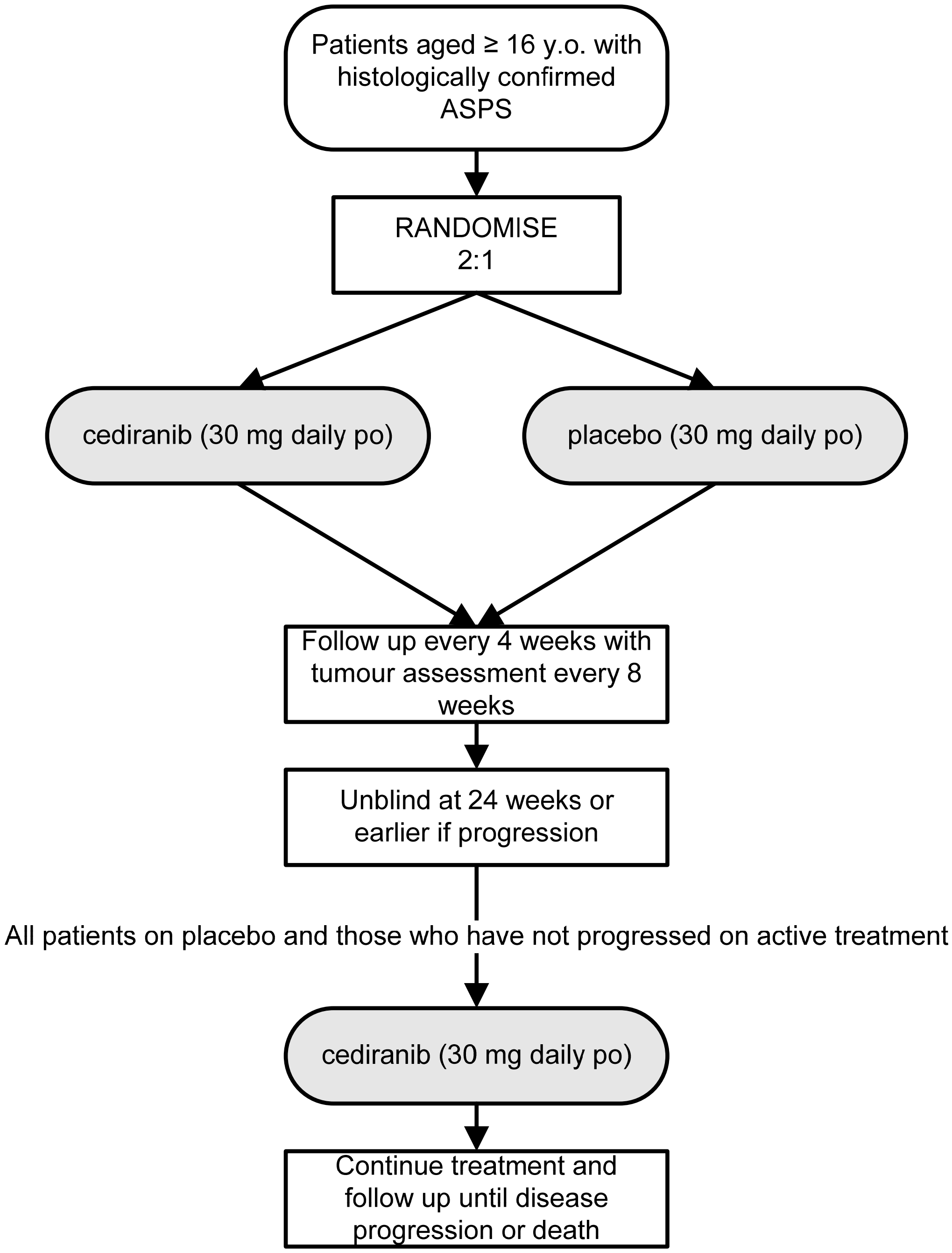

6. PATIENT SELECTION & ELIGIBILITY

6.1. Number of Patients

Thirty-six patients will be recruited into the study.

6.2. Source of Patients

As ASPS is a rare histological subtype of soft tissue sarcoma, it is acknowledged that there are a limited number of eligible patients. In UK centres, it is expected that there will be only 5-6 new cases per year eligible for trial participation. Therefore, patients will be recruited from a number of countries internationally. The combined population of participating countries closely approaches that of the USA. Therefore, it is anticipated that a recruitment rate of 36 patients will be achievable.

6.3. Inclusion Criteria

1. Histologically confirmed diagnosis of ASPS (central confirmation not required at study entry)
2. Age 16 years and older
3. Availability of archived tissue blocks to enable confirmation of t(X;17) translocation
4. ECOG Performance Status of 0-1
5. Life expectancy of >12 weeks
6. Progressive disease as defined by RECIST v1.1 within 6 months prior to randomisation
7. Measurable metastatic disease using RECISTv1.1, i.e. at least one lesion 10 mm in diameter (15 mm in short axis for nodal lesions) assessable by CT (or MRI for brain metastases).
8. Patients with brain metastases are permitted provided disease is controlled with a stable dose of corticosteroid and/or non-enzyme inducing anticonvulsant
9. The capacity to understand the patient information sheet and ability to provide written informed consent
10. Willingness and ability to comply with scheduled visits, treatment plans, laboratory tests and other study procedures
11. Able to swallow and retain oral medication

6.4. Exclusion Criteria

1. Inadequate bone marrow reserve as demonstrated by an absolute neutrophil count ≤1.5 x 10^9/L or platelet count ≤100 x 10^9/L
2. Serum bilirubin ≥ 1.5 x ULN (unless Gilbert's syndrome)
3. ALT or AST ≥ 2.5 x ULN. If liver metastases are present, ALT or AST > 5 x ULN
4. Serum creatinine > 1.5 x ULN or a creatinine clearance (calculated or measured) of ≤ 50mL/min

5. Greater than +1 proteinuria unless urinary protein < 1.5g in a 24 hr period or protein/creatinine ratio < 1.5.
6. History of significant gastrointestinal impairment, as judged by the Investigator, that would significantly affect the absorption of cediranib.
7. Patients with a history of poorly controlled hypertension with resting blood pressure >150/100 mmHg in the presence or absence of a stable regimen of anti-hypertensive therapy.
8. Any evidence of severe or uncontrolled co-morbidities e.g. unstable or uncompensated respiratory, cardiac, hepatic or renal disease, or active and uncontrolled infection.
9. Evidence of prolonged QTc ≥480 msec (using Bazetts correction, for which the formula is: $QTc = QT/\sqrt{RR}$) or history of familial long QT syndrome.
10. Significant recent haemorrhage (>30mL bleeding/episode in previous 3 months) or haemoptysis (>5mL fresh blood in previous 4 weeks).
11. Major thoracic or abdominal surgery in the 14 days prior to entry into the study, or a surgical incision that is not fully healed.
12. Pregnant or breast-feeding women; women of childbearing potential with a positive pregnancy test prior to receiving study medication; women the intention of pregnancy during study treatment; women of child bearing potential unwilling to have a urine or serum pregnancy test prior to study entry (even if surgically sterilised).
13. Men and women of childbearing potential unwilling to use adequate birth control measures (e.g. abstinence, oral contraceptives, intrauterine device, barrier method with spermicide, implantable or injectable contraceptives or surgical sterilisation) for the duration of the study and should continue such precautions for 2 weeks after receiving the last study treatment.
14. History of anticancer (including investigational, non-registered) treatment in the four weeks prior to first dose of cediranib, with the exception of palliative radiotherapy for symptom control.
15. Previous treatment with cediranib.
16. Known hypersensitivity to any excipient of cediranib.
17. History of other malignancies (except for adequately treated basal or squamous cell carcinoma or carcinoma in situ) within 5 years, unless the patient has been disease free for 2 years and there is a tissue diagnosis of the primary cancer of interest from a target lesion.
18. Other concomitant anti-cancer therapy (including LHRH agonists) except steroids
19. Recent history of thrombosis
20. Patients with brain metastases if they are symptomatic requiring increasing steroids in the previous six weeks to study entry or those with evidence of recent and/or active bleeding, or those causing uncontrolled seizures.

7. CONSENT

7.1. Procedure for Obtaining Informed Consent

The Principal Investigator (or designee) should discuss the trial with potentially eligible patients, describing the purpose, alternatives, drug administration plan, research objectives and follow-up of the study. Patients will be provided with an ethically approved patient information sheet and consent form for review and given sufficient time to consider participation in the study. Once a decision has been made to enter into the trial, a signature should be obtained from the patient to confirm consent. Consent should be obtained before any assessments prescribed by the protocol are performed.

Once a patient has consented to study participation a Registration Form should be faxed to the ICR-CTSU **(+44 (0)20 8770 7876)** so that the trial management team are informed that a patient has entered screening.

8. SCREENING

8.1. Clinical Evaluations

Investigator(s) must keep a record of subjects who entered screening but were not randomised on a subject screening log, including a reason for their screen failure.

The following screening evaluations should be performed to confirm eligibility for the trial. No trial specific procedures will be carried out prior to provision of written informed consent:

Within 30 days prior to the subject starting study drug:

1. CT scan (or MRI for brain metastases) and tumour assessment. Please send an anonymised copy of this scan to ICR-CTSU for central review.

Within 14 days prior to the subject starting study drug:

1. Physical examination (including vital signs, height and weight). BP measurements will be made after the patient has been resting in the supine position for a minimum of 5 minutes. Two or more readings should be taken at 2-minute intervals and averaged. If the first 2 diastolic readings differ by more than 5 mmHg, then an additional reading should be obtained and averaged.
2. ECOG performance status
3. Review of current medications; medical history; baseline symptoms and demographics.

Within 7 days prior to the subject starting study drug:

1. Laboratory investigations including full blood count, serum chemistry, (including AST, ALT, GGT, ALP, total bilirubin, bicarbonate, creatinine, urea, Na^+, K^+, Mg^{2+}, Ca^{2+}, Cl^-, glucose, LDH and amylase, phosphate, total proteins and albumin), urinalysis (24 hours urine collection if dipstick shows protein to be 2+) and thyroid function tests (TSH, free thyroxine [T4])

Within 72 hours prior to the subject starting study drug:

1. A resting 12-lead electrocardiograph (ECG) will be performed (duplicate). Further ECGs will be performed when clinically indicated, e.g. in the event of a cardiac AE. Any clinically significant abnormal findings observed and recorded during the study will be recorded as an AE.
2. Urine or serum pregnancy test in women of childbearing potential

8.2. Pathology Review

A tumour block of primary tissue from the time of diagnosis will be required from each study subject to confirm diagnosis, and availability of this is an inclusion criteria for the study. Tissue blocks from a known recurrence will be accepted if the original tumour samples are unavailable. The diagnosis of ASPS will be reviewed centrally by the Department of Histopathology of the Royal Marsden Hospital and evidence for the presence of a t(X;17) translocation will be investigated centrally by the Molecular Diagnostics Department at the Royal Marsden Hospital. Confirmation of diagnosis by central review is not required prior to trial entry.

8.3. Optional Research Samples (see Laboratory Manual for methods)

Within 1 month prior to the subject starting study drug:

1. Optional (if separate consent obtained, and tumour is amenable to biopsy) pre-treatment tumour biopsies (see Laboratory Manual for further detail).

Within 1 week prior to the subject starting study drug:

Optional (if separate consent obtained) pre-treatment samples:

1. Two 5 mL blood samples in CTAD tubes for determination of angiogenic factors.
2. Two 4 mL CPT tubes containing sodium citrate for peripheral blood mononuclear cells separation for later endothelial and rare cells isolation.
3. One 2.5 mL Paxgene Tube for nucleic acid isolation.

9. RANDOMISATION

9.1. Randomisation Procedure

Following written informed consent and completion of all screening procedures, an eligibility checklist should be completed by the Investigator to confirm patient eligibility for the trial. Patients can then be randomised to study treatment.

All randomisations will be done centrally by ICR-CTSU, in the UK.

Blank randomisation forms will be provided to sites with the Investigator Site File prior to study initiation. The following information will be requested in the randomisation form:

- Name of centre, Principal Investigator and person randomising patient
- Confirmation that written informed consent has been obtained
- Patient's full name (if applicable) and date of birth
- Confirmation that the patient is eligible and that an eligibility checklist has been completed

To randomise a patient
**Fax** the completed randomisation form to **+44 (0)20 8770 7876**<br><br>The randomisation desk will be open from 9.00 to 17.00 GMT therefore **you must allow 24 hours** for confirmation of randomisation and treatment allocation to be returned.

Within 24 hours, ICR-CTSU will return the completed randomisation form which will provide a unique randomisation number for the patient (Trial ID) together with the allocated treatment code. This fax confirmation will also be sent to the appropriate pharmacist to enable dispensing of the study treatment.

10. SCHEDULE OF ASSESSMENTS

	Screening	Baseline / Cycle 1 Day 1	Wk 1 Cycle 1 Day 8	Wk 2 Cycle 1 Day 15	Wk 3 Cycle 1 Day 22	Wk 4	Wk 8	Wk 12	Wk 16	Wk 20	Wk 24	Wk 32 – 48 (8 weekly) Wk 48 – cont (12 weekly)	Treatment discontinuation	Safety Follow Up (30 days post treatment discontinuation)	Annual Survival Follow-Up
Written Informed consent	X[a]														
Inform ICR-CTSU that pt entered screening	X[b]														
Randomisation		X[h]													
Study drug dispense [j]		X				X	X	X	X	X	X	X			
Treatment compliance and accountability						X	X	X	X	X	X	X	X		
Treatment unblinding											X [n]				
Demographics	X[e]														
Medical history	X[e]														
Concurrent meds	X[e]	X[i]		X		X	X	X	X	X	X	X	X	X	
Adverse events		X[i, k]		X		X	X	X	X	X	X	X	X	X	
BP Monitoring [p]	X[e]	X[i]	X[l]	X	X[l]	X	X	X	X	X	X	X	X		
Physical exam/vital signs/weight/ ECOG performance status	X[e]	X[i]		X		X	X	X	X	X	X	X	X	X	
Height	X[e]														
Full Blood Count [q]	X[f]			X		X	X	X	X	X	X	X	X	X	
Serum Chemistry[r]	X[f]			X		X	X	X	X	X	X	X	X	X	
Pregnancy Test	X[g]														
Thyroid function	X[f]					X	X		X		X	X		X	
Urine dipstick or urinalysis for protein	X[f]			X		X	X	X	X	X	X	X	X	X	
ECG [s]	X[g]			X[m]		X[m]									
CT (or MRI) and Tumour Assessment (RECISTv1.1) Send CD for central review of Choi response[t]	X[d]						X		X		X	X			
Additional blood (research) samples[c,u,v]	X[f]	X		X		X	X						X		
Tumour Biopsies (optional)[c,w]	X[d]			X									X[o]		
Survival															X

a: Prior to any study specific assessments or procedures
b: Send Registration Form to ICR-CTSU following consent of a patient to enter study screening
c: If separate written informed consent is obtained
d: Within 30 days prior to starting study treatment
e: Within 14 days prior to starting study treatment
f: Within 7 days prior to starting study treatment
g: Within 72 hours prior to starting study treatment
h: Can be done prior to Day 1 as screening procedures timed with start of treatment
i: Prior to study drug administration
j: Continuous dosing from Day 1 Cycle 1 of 30 mg OD, PO until confirmation of disease progression
k: Adverse Events to be collected from time of randomisation
l: Can be performed at local GP practise or with portable BP monitoring system at home
m: 2 hours post dose
n: All patients remaining progression free should be allowed to receive open-label cediranib from 24 weeks
o: At disease progression
p: In the event of hypertension, refer to hypertension management plan for monitoring frequency
q: Hb, WBC and differential, platelets
r: Sodium, potassium, magnesium, urea, creatinine, alkaline phosphatase, ALT, AST, GGT, total protein, albumin, total bilirubin, bicarbonate, calcium, chloride, glucose, LDH, amylase, phosphate
s: As specified, and as clinically indicated.
t: The schedule of CT/MRI scans will not re-start for patients switching from placebo to cediranib following systematic unblinding. i.e., For all patients scans are every 8 weeks from Baseline in the first 12 months and 12-weekly thereafter. Please send a copy of the anonymised CT scan to ICR-CTSU taken at screening, Week 24 and at time of disease progression.
u: Two per 5 ml CTAD tubes, 2 per 4 mL CPT tubes containing sodium citrate, 1 per 2.5 mL Paxgene Tube.
v: The schedule of additional blood (research) samples will not re-start for patients switching from placebo to cediranib following systematic unblinding. i.e., Baseline, Weeks 2, 4 and 8 blood samples are not re-taken when patients start open-label cediranib.
w: For patients switching from placebo to cediranib who have consented to an additional biopsy following unblinding, the additional biopsy will be taken at Week 2 of open-label cediranib treatment.

11. TRIAL ASSESSMENTS

11.1. Baseline (and / or Cycle 1 Day 1) (Where 1 cycle is 28 days)

Pre-treatment administration

1. Review of current medications*
2. Physical examination (including vital signs and weight), review of any new symptoms or adverse events*
3. Optional (if separate consent obtained) research blood samples*, see Section 11.4
4. Treatment allocation and dispensing

11.2. On-treatment Assessments

11.2.1. Week 1 (Cycle 1 Day 8, +/- 2 days)

1. Blood pressure monitoring (performed at GP practise or with portable BP machine at home)

11.2.2. Week 2 (Cycle 1 Day 15, +/-1 day)

Pre-dose

1. Physical examination (including vital signs, weight and ECOG performance status)
2. Review of adverse events
3. Review of current medications
4. Laboratory investigations: full blood count, serum chemistry (including AST, ALT, GGT, ALP, total bilirubin, bicarbonate, creatinine, urea, Na^+, K^+, Mg^{2+}, Ca^{2+}, Cl^-, glucose, LDH and amylase, phosphate, total proteins and albumin), urine dipstick or urinalysis (proteinuria in 24 urine collection if two consecutive 2+ or one 3+ or greater proteinuria assessments)
5. Optional research blood samples, see Section 11.4

Post-dose

6. Optional (if separate consent obtained and tumour is amenable to biopsy) Day 15 (+/- 1 day) post-treatment tumour biopsy (see Laboratory Manual for further detail)
7. ECG 2 hours (+/- 30 minutes) post-dose

11.2.3. Week 3 (Cycle 1 Day 22, +/- 2 days)

1. Blood pressure monitoring (performed at GP practise or with portable BP machine at home)

11.2.4. Week 4 (Cycle 2 Day 1, +/- 2 days)

Pre-dose

1. Physical examination (including vital signs, weight and ECOG performance status)
2. Adverse events, grade and causality relationship
3. Review of current medications
4. Laboratory investigations: full blood count, serum chemistry (including AST, ALT, GGT, ALP, total bilirubin, bicarbonate, creatinine, urea, Na^+, K^+, Mg^{2+}, Ca^{2+}, Cl^-, glucose, LDH and amylase, phosphate, total proteins and albumin), urine dipstick or urinalysis (proteinuria in 24 urine collection if two consecutive 2+ or one 3+ or greater proteinuria assessments)
5. Thyroid function test (TSH, free thyroxine [T4])
6. Optional research blood samples, see Section 11.4

Post-dose

7. ECG 2 hours (+/- 30 minutes) post-dose

11.2.5. Weeks 8-24, every 4 weeks (+/- 3 days); Weeks 24-48, every 8 weeks (+/- 3 days); Week 48 onwards, every 12 weeks (+/- 1 week)

1. Physical examination (including vital signs, weight and ECOG performance status.
2. Laboratory investigations: full blood count, serum chemistry (including AST, ALT, GGT, ALP, total bilirubin, bicarbonate, creatinine, urea, Na^+, K^+, Mg^{2+}, Ca^{2+}, Cl^-, glucose, LDH and amylase, phosphate, total proteins and albumin), urine dipstick or urinalysis (proteinuria in 24 urine collection if two consecutive 2+ or one 3+ or greater proteinuria assessments)
3. Review of adverse events
4. Review of concurrent medications
5. Assessment of compliance with study medication

11.2.6. Weeks 8 – 48, every 8 weeks (+/- 3 days); Week 48 onwards, every 12 weeks (+/- 1 week)

1. Thyroid function test (TSH, free thyroxine [T4])
2. Optional research blood sample at 8 weeks only, see Section 11.4
3. ECG, if clinically indicated

11.2.7. Weeks 8 – 48, every 8 weeks (+/- 3 days); Week 48 onwards, every 12 weeks (+/- 1 week). *NB. This schedule will not re-start for patients swopping from placebo to cediranib at 24 weeks.*

1. CT or MRI scan and tumour evaluation according to RECISTv1.1 criteria

2. Copy of anonymised scan sent for central assessment of radiological response by Choi criteria in the CT scan or MRI at Week 24

11.3. Treatment Discontinuation (for all patients at the time of discontinuation of treatment).

1. Physical examination (including vital signs, weight and ECOG performance status)
2. Review of adverse events
3. Review of current medications
4. Assessment of compliance with study medication
5. Laboratory investigations: full blood count, serum chemistry (including AST, ALT, GGT, ALP, total bilirubin, bicarbonate, creatinine, urea, Na^{+}, K^{+}, Mg^{2+}, Ca^{2+}, Cl^{-}, glucose, LDH, amylase, phosphate, total proteins and albumin), urine dipstick or urinalysis (proteinuria in 24 urine collection if two consecutive 2+ or one 3+ or greater proteinuria assessments)
6. Optional research blood samples, see Section 11.4
7. Optional (if separate consent obtained and tumour is amenable to biopsy) tumour biopsy taken if treatment is discontinued because of disease progression (see Laboratory Manual for further detail)
8. Copy of anonymised scan sent for central assessment of radiological response by Choi criteria in the CT scan or MRI.

11.4. Optional Research Sample Collection (see Laboratory Manual for detail and methods)

Tissue samples

If patients have solid tumours which are amenable to biopsy, consent should be sought to take pre- (within 1 month of Cycle 1 Day 1) and post-treatment biopsies (Cycle 1 Day 15 or as soon as feasible), and at the time of disease progression in order to examine the effect of cediranib on gene expression and tumour vascularity. For patients switching from placebo to cediranib following systematic unblinding who have provided separate consent to optional tissue samples, an additional biopsy should be taken at Week 2 (Cycle 1 Day 15 or as soon as feasible) after starting open-label cediranib.

Blood samples

The following optional blood samples will be taken, if separate consent is obtained pre-dose at Screening (within 7 days of starting treatment), Baseline (Cycle 1, Day 1 prior to treatment start), Week 2, Week 4, Week 8 and at the End of Treatment visit:

- Two 5 mL blood samples in CTAD tubes for determination of angiogenic factors.

- Two 4 mL blood samples CPT tubes containing sodium citrate for peripheral blood mononuclear cells separation and later endothelial and rare cells isolation.
- One 2.5 mL Paxgene Tube for nucleic acid isolation.

12. UNBLINDING

12.1. Procedure for Systematic Unblinding of all Patients

On completion of 24 weeks of treatment, all patients should be unblinded. The site should contact ICR-CTSU by faxing an unblinding request form to +44 (0)20 8770 7876. The unblinding request form should only be sent when all Week 24 study assessments have been completed – including tumour evaluation according to RECIST v1.1 criteria. ICR-CTSU will return this form to the local pharmacy and the Principal Investigator informing them of the blinded treatment the patient was receiving.

Patients who have been receiving cediranib, who remain progression free, should continue cediranib treatment until disease progression.

Patients who have been receiving placebo will be allowed to switch to open label cediranib treatment. These patients will re-start the study visit schedule from Cycle 1 Day 1 in order to ensure the safety of patients on cediranib. However, the CT/MRI scan schedule will not restart but remain on the same schedule (8 weekly from Baseline in the first 12 months, and 12 weekly thereafter). A CT/MRI scan and tumour evaluation according to RECISTv1.1 criteria must be done within 30 days prior to starting open-label cediranib.

The schedule of additional blood (research) samples will not re-start for patients switching from placebo to cediranib following systematic unblinding. i.e., Baseline, Weeks 2, 4 and 8 samples will not be re-taken when patients start open-label cediranib having received placebo in the blinded phase.

For patients switching from placebo to cediranib following systematic unblinding an additional biopsy will be taken at Week 2 of open-label cediranib treatment.

Patients will continue cediranib treatment until disease progression.

12.2. Emergency unblinding

If in the first 24 weeks of treatment unblinding is required due to a medical emergency, code break information will be available on site. See section 16.8.

13. TREATMENT DURATION AND DISEASE PROGRESSION

Patients will continue study treatment until disease progression or death. Disease progression is defined by RECIST 1.1 criteria (Appendix 1).

If a patient has progressive disease in the first 24 weeks, they should be unblinded by faxing a request for unblinding form to ICR-CTSU as described in Section 12. The unblinding request form should only be sent when all study assessments for the patient's last blinded treatment cycle have been completed – including tumour evaluation according to RECIST v1.1 criteria. If the patient is confirmed as receiving cediranib they will discontinue study drug and be followed up as decribed in Sections 11.3, 14 and 15. If the patient is confirmed as receiving placebo treatment they will be allowed to switch to cediranib treatment. These patients will re-start all study procedures from Cycle 1 Day 1 (see Section 11).

If the Investigator is in doubt as to whether progression has occurred, particularly with response to a NTL (non-target lesion) or the appearance of a new lesion, it is advisable to continue treatment until the next scheduled assessment or sooner if clinically indicated and reassess the patient's status. If repeat scans confirm progression, then the date of the initial scan should be declared as the date of progression.

To achieve 'unequivocal progression' on the basis of non-target disease, there must be an overall level of substantial worsening in non-target disease such that, even in presence of SD or PR in target disease, the overall tumour burden has increased sufficiently to merit discontinuation of therapy. A modest 'increase' in the size of one or more non-target lesions is usually not sufficient to qualify for unequivocal progression status.

Patients who withdraw from treatment early at any point in the study (i.e. due to unacceptable toxicity) will have a Treatment Discontinuation visit as per Section 11.3 and a Safety Follow Up visit as per Section 14. Patients will continue to be followed up with regular scans as per the study schedule until documentation of disease progression, unless consent for further follow up is withdrawn.

14. SAFETY FOLLOW UP (30 DAYS AFTER TREATMENT DISCONTINUATION)

Patients will be followed for potential toxicities until 30 days after treatment discontinuation or in case of drug-related adverse events until their resolution.

1. Physical examination (including vital signs, weight and ECOG performance status)
2. Review of adverse events
3. Review of current medications

4. Laboratory investigations: full blood count, serum chemistry (including AST, ALT, GGT, ALP, total bilirubin, bicarbonate, creatinine, urea, Na^{+}, K^{+}, Mg^{2+}, Ca^{2+}, Cl^{-}, glucose, LDH, amylase, phosphate, total proteins and albumin), urine dipstick or urinalysis (two consecutive 2+ or one 3+ or greater proteinuria assessments)

15. FOLLOW UP

Following treatment completion, patients will be followed for survivalannually.

16. TRIAL TREATMENT

16.1. Drug Dose

Patients will be prescribed 30 mg cediranib (or matching placebo) daily. A number of studies conducted by AstraZenenca have identified 30 mg as the recommended dose for monotherapy, with sustained efficacy being reported at that dose. Lower dose tablets (20 mg) are available if required for a dose reduction, however dose interruptions should be used as the first approach to managing toxicity (see Section 17.1).

16.2. Drug Schedule

A treatment cycle is 28 days. At each visit, patients will be dispensed enough cediranib (or matching placebo) tablets (with adequate overage) to last until their next visit. In the first 24 weeks this will be a 28 day supply; between the 24 week and 48 week visits this will be an 8 week supply and from 48 weeks onwards this will be a 12 week supply. Patients will continue treatment until disease progression or withdrawal from treatment for any other reason.

Patients should take one 30 mg cediranib or two 15 mg cediranib (or matching placebo) tablets orally once a day. Patients should take their tablets on an empty stomach (either 1 hour before or 2 hours after meals). The tablets should be taken at approximately the same time each day. Tablets must be swallowed whole and not crushed.

If a patient forgets to take a dose, and it is within 6 hours of the scheduled time, then the patient should be advised to take them as soon as possible. If it is more than 6 hours after the scheduled time, then study medication should not be taken for that day. Study medication should continue as scheduled on the subsequent day. A patient should not take more than a single day’s dose of tablets within a day.

In the event that the patient cannot hold the tablets down (if the patient vomits) within 30 minutes from taking the tablets and the tablets can be identified in the vomit content, the patient can re-take new tablets from the bottle.

16.3. Drug Manufacturer

Cediranib (also known as AZD2171 maleate and Recentin™) and matching placebo are manufactured and provided free of charge by AstraZeneca to participating centres.

16.4. Drug Presentation, Packaging and Labelling

Cediranib and matching placebo are presented as 30 mg (9 mm), 20 mg (8 mm) or 15 mg (7 mm), beige film-coated tablets and supplied in HDPE (Heavy Duty Polyethylene) bottles, with child resistant caps and tamper evident induction seals. All tablet bottles contain 35 tablets, providing a 7 day 'overage' to allow for visit delays.

To ensure that the patient and research team remain blind to the treatment allocation during the first 24 weeks of treatment, the drug label will be identifiable by means of a coded number. Matching code-break envelopes will be supplied with study drug which should be kept in pharmacy with restricted access, and used only in an emergency.

AstraZeneca have employed Fisher Clinical Services to coordinate IMP packaging, labelling and distribution for this trial. The schedule of responsibilities is detailed in a contract between AstraZeneca and Fisher Clinical Services, a three part Quality Agreement between AstraZeneca, Fisher Clinical Services and the Co-Sponsors details the schedule of responsibilities for this arrangement.

Labelling (including translations) will be the responsibility of Fisher Clinical Services and will be compliant with Annex 13 of the Good Manufacturing Guidelines (GMP) and all applicable local regulatory requirements.

16.5. Drug Distribution

No drug will be distributed to participating centres unless ICR-CTSU is satisfied that the required approvals and agreements and initiation procedures are complete.

Participating pharmacy departments should contact Fisher Clinical Services via the ICR-CTSU trial management team to request drug supplies. Contact details will be provided in trial specific guidance documents. Management of study drug will be coordinated by ICR-CTSU in the UK and, as such, contact will be between the hours of 9.00 and 17.00 GMT. Once a supply request is received, delivery will take up to 5 days within Europe and up to 10 days outside Europe.

Records must be kept for all deliveries and a copy of the order / delivery note placed in the Site Pharmacy File and kept within the pharmacy department as in routine practice. At the

end of the trial, it must be possible to reconcile supply and usage of all stock. Account must be given to any discrepancies and certificates of delivery and return must be signed.

Participating pharmacy departments will have an initial supply of lower dose drug (20 mg tablets). If dose reductions are required due to treatment toxicities as detailed in Section 17, centres should inform the ICR-CTSU.

16.6. Drug Storage

Cediranib and matching placebo should be stored in the original package at controlled room temperature (between 15°C and 30°C). Participating centres will be responsible for reporting any temperature excursions from these storage conditions via ICR-CTSU, to AstraZeneca for approval. Cediranib has a total shelf life of 4 years, but expiry dates will be clearly labelled on each individual bottle.

The local pharmacy is responsible for ensuring that the study medication is stored appropriately and in a secured area.

16.7. Drug Compliance and Accountability

The study drug must not be used outside the context of the CASPS protocol.

Patients must be asked to bring all their trial medication every time they attend the clinic, for the purposes of treatment compliance assessment and drug accountability. Every effort should be made to encourage patients to return the unused medication and empty packs/bottles. The unused tablets should be collected by the investigator/study nurse and counted to ascertain patient compliance, medication will then be returned to pharmacy for drug accountability prior to destruction according to local practices. Drug accountability and destruction records should be maintained by the local pharmacy.

16.8. Emergency Code Breaking

Treatment is blinded during the first 24 weeks of the study. During this time, in the absence of disease progression, unblinding is restricted to emergency situations and should only be used under circumstances where knowledge of the treatment is necessary for the proper clinical care of the patient. Each hospital pharmacy will be issued with code break envelopes and information on how to code break. The code must only be broken by the pharmacist. Details divulged to the clinician must only be in regard to specific treatment for the patient for whom the code is broken.

Emergency code break envelopes should not be used for the systematic unblinding at the 24 week visit. The ICR-CTSU should be contacted at this time as described in Section 12.1.

16.9. Duration of Trial Treatment

Treatment should continue until confirmed disease progression, death or withdrawal from treatment (i.e. due to unacceptable toxicity).

17. TOXICITY MANAGEMENT

Common toxicities listed in the Investigator Brochure include fatigue, diarrhoea, weight loss, anorexia, headache, nausea, vomiting, hoarseness, hand-foot syndrome and hypertension. Toxicities should be treated with maximum supportive care.

17.1. Dose Delays/Modifications

With the **exception** of the events detailed in sections 17.2–17.16, the following management plan should be followed for management of toxicity attributable to cediranib.

Dose interruptions should be used as the first approach to managing toxicity. For CTC Grade 3 or more, dosing should be interrupted, except in cases of nausea and vomiting which has not been managed with an adequate supportive treatment. For patients who have multiple moderate (grade 2) adverse events (for example, diarrhoea, weight loss, dehydration and fatigue) short dose interruptions (i.e. 2-5 days) may be considered. Treatment can be restarted on resolution at the same dose. Longer dose interruptions until toxicity recovery may be required for patients with chronic and moderate (grade 2) nausea and vomiting, diarrhoea, hand and foot syndrome and fatigue (with PS deterioration) if they are refractory to an adequate supportive treatment.

Cediranib dosing may be withheld for up to 14 days for management of toxicity. If a longer interruption is required due to unresolved toxicity, cediranib should be discontinued or a dose reduction to 20 mg considered.

Cediranib should be permanently discontinued in patients with the following conditions:

- Gastrointestinal perforation or wound dehiscence requiring medical intervention
- Serious haemorrhage, ie, requiring medical intervention
- Severe hypertension (see Appendix 3: Hypertension Management Guidelines)
- Nephrotic syndrome
- Severe arterial thromboembolic event
- Pregnancy

Adverse Events that are not covered by the explicit guidance provided in Section 17, including clinically significant abnormal laboratory results, should be managed according to local practise and with due regard to their severity.

17.2. Hypertension

Cediranib therapy has been associated with increases in blood pressure (BP) and cases of mild to moderate hypertension. CTC Grade 4 hypertension events (hypertensive crisis, end organ damage) have been rare and are manageable with standard anti-hypertensive therapy, such as dihydropyridine derivatives and angiotensin-converting enzyme inhibitors. Pre-existing cardiovascular risks should be assessed and managed and pre-existing hypertension should be adequately controlled before starting treatment with cediranib.

Blood pressure should be monitored from the start of treatment with cediranib and frequently thereafter. Detailed hypertension management guidelines are contained in Appendix 3 for the proactive medical management of emergent hypertension. In case of persistent or severe hypertension, despite optimal use of antihypertensive medicinal products and cediranib dose reduction, cediranib should be permanently discontinued

17.3. Posterior reversible encephalopathy syndrome

Posterior reversible encephalopathy syndrome (PRES) including reversible posterior leukoencephalopathy syndrome (RPLS) has been uncommonly reported in patients receiving cediranib in clinical studies. PRES, including RPLS, is a rare syndrome affecting vascular endothelial cells in the brain that may lead to capillary leak and oedema. It has been associated with a number of conditions, including renal failure, hypertension, fluid retention, and the use of cytotoxic or immunosuppressive drugs. It has also been reported rarely in association with the use of VEGF inhibitors including bevacizumab, sunitinib and sorafenib.

PRES can present in a variety of non-specific ways, including headache, seizures, lethargy, confusion, blindness and other visual and neurological disturbances, and can be fatal. Mild to severe hypertension may be present, but is not necessary for the diagnosis of PRES. Confirmation of PRES requires brain imaging, Magnetic Resonance Imaging (MRI) is the most sensitive imaging modality to detect PRES and is recommended in suspected cases to confirm the diagnosis. Cediranib should be discontinued following confirmation of PRES

In patients developing PRES, treatment of specific symptoms including control of hypertension is recommended. Active management of hypertension according to the hypertension management guidelines in Appendix 3 may reduce the incidence of PRES.

17.4. Diarrhoea

Cediranib is commonly associated with diarrhoea and therefore action should be taken to minimise its effects as soon as symptoms develop. Initial management of diarrhoea

- Patients should be made aware that they are likely to experience diarrhoea
- Patients should be given loperamide to take home with them
- If diarrhoea occurs patients should immediately start loperamide after the first episode (4mg initially then 2mg every 2 hrs) and continue to take it until they have been free from diarrhoea for at least 12 hrs
- Patients should be encouraged to drink plenty of fluids
- Patients should seek advice early, from their physician or study nurse, if
    - Grade 1 or 2 diarrhoea persists for over 24hrs despite treatment with loperamide
    - Grade 3 diarrhoea develops
    - Any grade of diarrhoea associated with vomiting, marked abdominal distension or inability to take oral fluids develops

Management of persistent (>24h) diarrhoea despite maximum treatment with loperamide

Patients should be advised to stop taking cediranib study tablets until resolution of diarrhoea and care should be taken to prevent dehydration. Following evaluation, consider antibiotics (for example an oral fluroquinolone for 7 days) particularly if the patient is neutropenic or has a fever. Consider infectious causes and aetiologies such as C-difficile/viral gastroenteritis. Cediranib may be restarted at the same dose once the patient has been free from diarrhoea for 12 hours.

Management of persistent (>48h) diarrhoea despite maximum treatment with loperamide

The physician or study nurse should see patients in this situation. Hospitalisation and IV fluids may be needed. Consider infectious causes and etiologies such as C.difficile/viral gastroenteritis. Consider antibiotics (for example an oral fluroquinolone for 7 days) particularly if the patient is neutropenic or has a fever. Octreotide (Sandostatin) may be considered.

In case of recurrent Grade 2 diarrhoea codeine phosphate could also be considered in combination with loperamide. Patients with persistent or severe diarrhoea (Grade 3 or higher) may require dose reduction to 20 mg or discontinuation of study medication. If CTC Grade 3 diarrhoea persists after 2 weeks, study medication should be discontinued.

17.5. Fatigue

Fatigue is very commonly reported during treatment with cediranib and onset may be rapid. During appointments, patients fatigue levels should be discussed. Care should be taken to ensure that the nutritional status of the patients is optimised and patients should be encouraged to drink plenty of fluids. Patients should be encouraged to manage fatigue by alternating periods of rest with light aerobic exercise, which may improve the symptoms in some cases. Consideration should be given to other possible causes (eg, thyroid dysfunction, depression/insomnia and other concomitant medications). If CTC Grade 2 fatigue with worsening in the ECOG performance status or grade 3 fatigue develops, patients should be advised to take short treatment breaks of the blinded study tablets (initially 2-3 days-or longer-up to a maximum of 14 days), in order to help relieve this symptom. Patients should restart treatment when symptoms have improved.

17.6. Hand and Foot syndrome

All patients will be encouraged to take action to prevent development of Hand and Foot syndrome including pedicure, wearing thick cotton gloves/socks, avoiding hot water and constrictive footwear or excessive friction. If grade 1 toxicity develops, patients could use moisturizing creams and 20%-40% urea cream for relief. If despite these measures toxicity increases to grade 2, consider transitory dose reduction to 50% for 7-14 days and add clobetasol 0.05% ointment, 2% lidocaine, codeine for pain. If grade 3 toxicity develops interrupt treatment for 7 days and consider re-starting treatment when toxicity has recovered to grade 1 and consider a permanent dose reduction to 20 mg.

17.7. Proteinuria

Proteinuria is reported very commonly in patients received cediranib although dose reductions are rarely required. During treatment, if patient has two consecutive two plus (++) proteinuria measurements on dipstick, or one three plus (+++) or greater measurement, a 24-hour urine specimen or protein/creatinine ratio sample should be collected. If 24-hour proteinuria is classified as CTC Grade 3 (4+ or 3.5g/24 hour) cediranib should be stopped until recovery to grade 1. If 24-hour proteinuria is classified as CTC Grade 4 (nephrotic syndrome), cediranib should be permanently discontinued.

17.8. Thrombocytopenia

Thrombocytopenia has been seen with monotherapy cediranib treatment. Patients with platelet count ≤100 x 109/L should not be included in studies with cediranib. All patients who have any CTC Grade 3 or 4 clinical laboratory values during a study or at the time of withdrawal from a study must have further tests performed until the values have returned to baseline, even if they have discontinued treatment, unless these values are not likely to improve because of the underlying disease.

17.9. Abnormal Liver Function

Increases in transaminases, which are sometimes associated with increases in total bilirubin, have been seen in cediranib clinical studies. All patients in whom significant abnormalities of AST, ALT, ALP, and total bilirubin of CTC Grade 3 or greater are documented, either during a study or at the time of withdrawal, cediranib should be discontinued until recovery to grade 1 has occurred. Further tests should be performed until the values have returned to baseline levels, even if they have discontinued treatment, unless these values are not considered unlikely to improve because of underlying disease progression.

17.10. Haemorrhage

VEGF inhibition is associated with increased risk of bleeding and although the underlying mechanism remains speculative bleeding events have been commonly reported following cediranib treatment. Mucocutaneous bleeding is the most frequent type of bleeding observed with gastrointestinal bleeding less frequent. Cediranib should be permanently discontinued in patients who have CTC Grade 2 and above haemorrhage.

17.11. Thyroid dysfunction: hypothyroidism and hyperthyroidism

Thyroid dysfunction has been commonly observed in patients treated with cediranib, which included dose-related increases from baseline in TSH levels sometimes associated with clinical hypothyroidism, and hyperthyroidism which was mostly mild/moderate and self-limiting. Patients have responded to replacement therapy without the need for stopping or reducing the dose of cediranib.

Patients with pre-existing thyroid dysfunction should be treated in accordance with standard medical practice prior to the start of therapy with cediranib.

Hypothyroidism could present at any time during cediranib treatment. Baseline laboratory measurement of thyroid function should be conducted in all patients and thyroid function should be monitored periodically throughout the course of treatment.

Replacement levothyroxine should be given when clinically indicated to normalise the thyroxine level to within the normal range, and before the patient becomes clinically symptomatic. Replacement levothyroxine therapy may also be considered in patients with TSH increases (and thyroxine levels within the normal range), together with adverse events and symptoms suggestive of incipient hypothyroidism. Thyroid function should be monitored frequently and the dose of levothyroxine should be titrated as required.

17.12. Renal Function

Nephrotoxicity is not an expected effect of cediranib treatment, however renal impairement could result secondary to hypertension. If Creatinine Clearance (measured or calculated) drops below 50 ml/min at any time during the study, study treatment should be suspended until recovery.

17.13. Arterial thromboembolism

Arterial thromboembolic events (including transient ischemic attack and ischemic stroke) have been reported in clinical studies with cediranib. Cediranib should be used with caution in patients who are at an increased risk of thrombotic events or who have a history of thrombotic events. Cediranib treatment should be permanently discontinued in patients who develop an arterial thromboembolic event.

17.14. Venous thromboembolism

Venous thromboembolic events including deep vein thrombosis and pulmonary embolism have been commonly reported in patients treated with cediranib. Anticoagulant treatment should be started in accordance with clinical practice. Discontinuation of cediranib may be considered. Cediranib should be used with caution in patients at risk of venous thromboembolism.

17.15. Gastrointestinal perforation

GI perforation has been uncommonly reported in patients treated with cediranib. Cediranib should be used with caution in patients at risk and permanently discontinued in patients who develop GI perforation.

17.16. Fistulae

In patients treated with cediranib, fistulae have been reported and reflected the location of the underling malignancy. Cediranib should be used with caution in patients at risk of fistula and discontinuation of cediranib should be considered in patients who develop a fistula.

18. TREATMENT PRECAUTIONS

18.1. Surgery during Cediranib Treatment

For elective surgery during the study, or any procedure that carries a risk of internal bleeding, it is recommended that cediranib be stopped for 2 consecutive weeks prior to the surgical procedure. Cediranib treatment can be restarted when the surgical wound has healed. If emergency surgery is performed, precautions should be taken to minimise the potential risk of bleeding and thrombosis associated with this class of agents, cediranib should be stopped and close monitoring for bleeding, wound healing and thromboembolic

complications should be initiated. Patients should not receive cediranib within 2 weeks of major abdominal or thoracic surgery.

18.2. Pregnancy and Breast Feeding

Cediranib should not be administered to pregnant or breast feeding women. A negative pregnancy test must be confirmed before administration of cediranib to women of childbearing potential. Female patients must be post-menopausal, surgically sterile, sexually abstinent or use two reliable forms of contraception from starting cediranib and for 2 weeks after the last dose. Reliable methods of contraception should be used consistently and correctly, acceptable methods include barrier methods, implants, injectables, combined oral contraceptive methods, some IUDs or vasectomised partner. Postmenopausal females are defined as:

- Natural menopause with menses >1 year ago
- Radiation-induced oophorectomy with last menses >1 year ago
- Chemotherapy-induced menopause with 1 year interval since last menses
- Serum follicle stimulating hormone, luteinising hormone and plasma oestradiol levels in the postmenopausal range for the institution
- Bilateral oophorectomy or hysterectomy

Male patients must use a barrier method of contraception from starting cediranib and for 2 weeks after the last dose.

18.3. Blood Donors

Patients who are blood donors should not donate blood during the study and for 3 months following their last dose of study treatment.

18.4. Bone Growth

Inhibition of angiogenesis will affect the development of growth plates in long bones. Young patients in whom epiphyseal growth plates have not closed should be made aware that cediranib might prevent future bone growth.

18.5. Concomitant Medications

All medication considered necessary for the patients' welfare and which is not expected to interfere with the evaluation of the study drugs may be given at the discretion of the investigator. Concomitant medications (including start/stop dates, dose frequency, route of administration and indication), must be recorded in the patient's source documentation, as well as the appropriate pages of the case report form.

The following treatment restrictions apply, for the safety of patients:

- Caution in the concomitant use of any medication that may markedly affect renal function (eg, vancomycin, amphotericin, ibuprofen, pentamidine). Such medications may, however, be used with caution if deemed essential for the treatment of a particular infection or continued if patients were using them prior to commencing the study with no effect on renal function demonstrable on blood or urine testing.
- Caution should be exercised in the concomitant use of any medication that may affect hepatic cytochrome P450 drug metabolising activity by way of enzyme induction (eg, phenytoin) or inhibition (eg, ketoconazole, ritonavir, erythromycin) within 2 weeks of the first dose of cediranib and throughout the study period.
- Patients who require oral anticoagulants (coumadin, warfarin) are eligible, provided there is increased vigilance with respect to monitoring INR. If medically appropriate and treatment available, consider switching to low molecular weight heparin.

19. PHARMACOVIGILANCE

19.1. Adverse Event Definitions

Adverse Event (AE): any untoward medical occurrence in a patient or clinical trial subject administered a medicinal product and which does not necessarily have a causal relationship with this treatment. *An AE can therefore be any unfavourable and unintended sign, symptom, or disease associated with the use of a study drug, whether or not considered related to the study drug. CTCAE v4.0 Grade 2 or above abnormal laboratory findings are considered to be clinically significant and should be recorded as adverse events. Signs and symptoms of metastatic disease, as determined by the local clinical investigator, are not adverse events.*

Adverse Reaction (AR): all untoward and unintended responses to the study drug related to any dose administered. *All AEs judged by either the reporting investigator or the sponsor as having reasonable causal relationship to a medicinal product qualify as adverse reactions i.e. an AR is possibly, probably or definitely related to the study drug. The expression reasonable causal relationship means to convey in general that there is evidence or argument to suggest a causal relationship.*

Serious Adverse Event (SAE) or Serious Adverse Reaction (SAR): Any untoward medical occurrence or effect which occurs within 30 days of the patient receiving study drug that at any dose:

- results in death: *the patient's death is suspected as being a direct outcome of the AE.*
- is life-threatening: *refers to an event in which the subject was at risk of death at the time of the event. It also refers to an event that would result in death with the*

continued use of the product; it does not refer to an event which hypothetically might have caused death if it were more severe.

- requires hospitalisation, or prolongation of existing inpatient hospitalisation: *admission to hospital overnight or prolongation of a stay in hospital was necessary as a result of the AE. Outpatient treatment in an emergency room is not itself an SAE, although the reasons for it may be. Hospital admissions/surgical procedures planned for a pre-existing condition before a patient is randomised to the study are not considered SAEs, unless the illness/disease deteriorates in an unexpected way during the study.*
- results in persistent or significant disability or incapacity: *the AE results in a significant or persistent change, impairment, damage or disruption in the patient's body function/structure, physical activities or quality of life.*
- is a congenital anomaly or birth defect
- Important medical events that may not be immediately life-threatening or result in death or hospitalisation but may jeopardise the patient or may require intervention to prevent one of the other outcomes listed in the definition above should also be considered serious.

N.B. progressive disease and death due to disease are not considered SAEs but should be reported on the relevant forms (i.e. progression form for relapse and death form for death).

Suspected Unexpected Serious Adverse Reaction (SUSAR): Any serious adverse event with a suspected relationship to study drug that is not listed on the Investigator Brochure and, in the opinion of the Chief Investigator, is unexpected.

19.2. Causality (Relationship to Study Drug)

Many adverse events that occur in this trial, whether they are serious or not, will be known treatment related toxicities. The Principal Investigator is responsible for the assessment of causality of serious adverse events (see definitions of causality table).

If there is any doubt about the causality of an event, the investigator should inform ICR-CTSU who will notify the Chief Investigator. ICR-CTSU of the Chief Investigator may contact the drug manufacturer and/or other clinicians if specific advice or further information is required.

Definitions for Causality

Relationship	Description
**Unrelated**	There is no evidence of any causal relationship with the trial drug
**Unlikely**	There is little evidence to suggest there is a causal relationship (e.g. the event did not occur within a reasonable time after administration of the trial medication). There is another reasonable explanation for the event (e.g. the patient's clinical condition, other concomitant treatment)
**Possible**	There is some evidence to suggest a causal relationship (e.g. because the event occurs within a reasonable time after administration of the trial medication). However, the influence of other factors may have contributed to the event (e.g. the patient's clinical condition, other concomitant treatments)
**Probable**	There is evidence to suggest a causal relationship, and the influence of other factors is unlikely
**Definitely**	There is clear evidence to suggest a causal relationship, and other possible contributing factors can be ruled out

19.3. Expectedness

Adverse events which are expected to occur with cediranib treatment, according to previous clinical trials, are listed in the Investigator Brochure. The expectedness of an SAE will be assessed by the Chief Investigator (or the CIs delegate) in accordance with the information in the Investigator Brochure.

19.4. Reporting Serious Adverse Events (SAEs) to ICR-CTSU

Any SAE that occurs from randomisation to study treatment and up to 30 days following the last dose of study drug must be reported.

All SAEs should be reported to ICR-CTSU, within 24 hours of the Principal Investigator (or designated representative) becoming aware of the event, by completing the trial specific SAE forms and faxing to:

The ICR-CTSU safety desk

Fax no: +44 (0) 208 722 4368

For the attention of the CASPS Trial team

All SAE forms must be completed, signed and dated by the Principal Investigator or designated representative.

19.5. Pregnancy

Any new pregnancy that occurs during study participation must be reported using a clinical trial pregnancy form to the above number. To ensure subject safety, each pregnancy must be reported to ICR-CTSU within 72hrs of learning of its occurrence. The pregnancy must be followed up to determine outcome (including premature termination) and status of

mother and child. Pregnancy complications and elective terminations for medical reasons must be reported as an AE or SAE. Spontaneous abortions must be reported as an SAE.

Any SAE occurring in association with a pregnancy brought to the investigator's attention after the subject has completed the study and considered by the investigator as possibly related to the study treatment, must be promptly reported to ICR-CTSU.

In addition, the investigator must attempt to collect pregnancy information on any female partners of male study subjects who become pregnant while the subject is enrolled in the study. Pregnancy information should be reported to ICR-CTSU as described above.

19.6. Review of Serious Adverse Events (SAEs)

Reported SAEs will be assessed by the Chief Investigator (or designated representative) for causality and expectedness. *NB. The Chief Investigator cannot down grade the Principal Investigator's assessment of causality.*

SAEs assessed as having a causal relationship to study drug and as being unexpected (SUSARs) will undergo expedited reporting to the relevant authorities by ICR-CTSU or local representative (see Section 19.7 for details of SAE reporting).

Centres should respond as soon as possible to requests from the Chief Investigator or his designate (via ICR-CTSU or local representative) for further information that may be required for final assessment of the SAE.

19.7. Expedited Reporting of SUSARs

If an SAE is identified as being a SUSAR by the Chief Investigator expedited reporting will be initiated by ICR-CTSU. If a SUSAR is fatal or life threatening, the timeframe for reporting to the regulatory authorities is within 7 days of being notified of the event. For non-fatal or non-life threatening events the time frame for reporting is within 15 days of being notified of the event. ICR-CTSU will report any additional relevant information as soon as possible, or within 8 days of the initial report of a fatal/life threatening SUSAR.

ICR-CTSU will report SUSARs to:

- The UK Competent Authority (MHRA)
- The UK Main Research Ethics Committee (SUSARs originating in the UK only)
- The Eudravigilance Database (SUSARs originating in EU Member States only. UK and third country SUSARs are reported to Eudravigilance by the MHRA)
- The Sponsor
- AstraZeneca

- The Sarcoma Group and/or country Lead Investigator for centres outside of the UK not participating via a national Sarcoma Group in each participating country

ICR-CTSU will report SUSARs to local UK Investigators at regular intervals.

The Sarcoma Group in each participating country will report SUSARs, as per their local requirements, to:

- The national Competent Authority
- IECs
- Local Investigators

In all instances ICR-CTSU will require confirmation of onward reporting within specified timelines from the coordinating Sarcoma Group and/or country Lead Investigator for centres outside of the UK not participating via a national Sarcoma Group in each country.

19.8. Unblinding of SUSARs

Unblinding of SUSARs for regulatory reporting purposes will be done centrally at ICR-CTSU by the trial statistician to ensure that individual clinicians and staff directly involved in the conduct of the trial remain blinded to study treatment.

19.9. Follow up of Serious Adverse Events

Centres should continue to follow up SAEs until clinical recovery is complete and laboratory results have returned to normal, or until disease has stabilised. Information on outcome of the SAE should be completed on the relevant part of the original SAE form and faxed to ICR-CTSU as soon as the Principal Investigator becomes aware.

19.10. Annual Reporting of Serious Adverse Reactions (including SUSARs)

Annual reports will be submitted on the anniversary of the date when the Clinical Trial Authorisation was granted in each country. This will include a listing of all serious adverse reactions (including SUSARs), and a report from the joint Independent Data Monitoring Committee / Trial Steering Committee (IDMC/TSC).

ICR-CTSU will prepare the annual report as per local requirements, and provide these to:

- The MHRA
- The UK Main REC
- The Sponsor
- AstraZeneca

ICR-CTSU will prepare annual reports and send these to each participating Sarcoma Group and/or country Lead Investigator for centres outside of the UK not participating via a

national Sarcoma Group, so that these may be formatted according to local requirements and forwarded to:

- the Competent Authority
- IECs

in each participating country.

19.11. Flow diagram for SAE reporting, and action following report

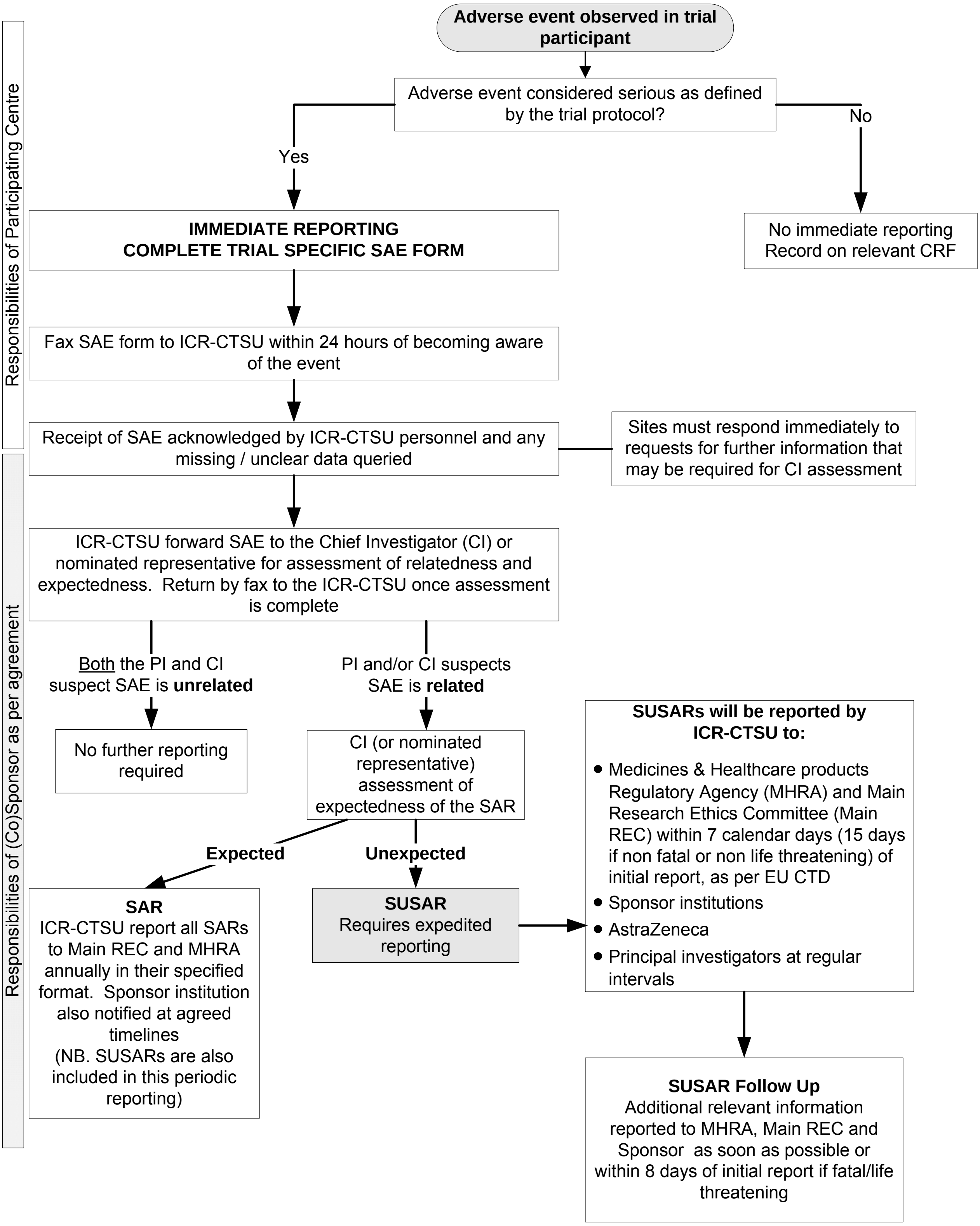

NB. All SAE's should continue to be followed up as specified in the protocol.

20. STATISTICAL CONSIDERATIONS

20.1. Trial Design

This is a 2-arm, randomised, double-blind phase II study of cedirinib versus placebo in the treatment of patients with metastatic ASPS which has progressed within the 6 months prior to randomisation.

20.2. Randomisation

Treatment allocation will be 2:1 (cedirinib/placebo). A randomisation list will be generated using random permuted blocks.

20.3. Primary Endpoint

The primary endpoint is week 24 (or progression if sooner) percentage change in the sum of target marker lesion diameters from randomisation.

20.4. Secondary Endpoints

Secondary endpoints are 24 weeks RECISTv1.1 response rate, best RECISTv1.1 response, best reduction (%) in tumour size, progression-free survival rate, percentage alive and progression-free at 12 months; overall survival rate and the safety and tolerability profile of cediranib in patients with ASPS.

20.5. Exploratory Endpoints

Exploratory endpoints will include radiological responses using Choi criteria, identification predictive angiogenesis markers of response, describe changes in angiogenesis markers and expression of angiogenesis regulatory genes in peripheral blood and optional tumour biopsies, explore changes in circulating endothelial cells and other rare cells events, including potential sarcoma circulating cells.

20.6. Statistical Background

The decision to perform a randomised study was based on the known tendency of ASPS to grow very slowly and, even when metastatic, to undergo periods of disease stabilisation. For this reason it is not possible to use progression-free survival as an endpoint without a comparator. Survival is typically prolonged, hence overall survival is not a feasible primary endpoint. The reason for choosing 24 weeks for the comparison between cediranib and placebo was similarly based on the period of time thought to be required to show a significant difference. Response could have been considered as the primary endpoint, but this assumes that activity similar to that observed in the pilot group of patients (AstraZeneca study D8480C00046) will always occur, which is probably not a sound assumption, owing to possible bias in patient selection.

20.7. Statistical Design

In the 6 patients treated in the AstraZeneca Study (D8480C00046), the mean tumour size reduction at first scan at 8 weeks was 25% with a coefficient of variation (CV) of 19%. By the time of the second scan at 16 weeks, 4 of 6 patients had a reduction in size of >30%, i.e. a partial response.

Assuming that a smaller effect and greater CV might be observed in a larger trial this study assumes a 20% reduction in the sum of target marker lesion diameters and CV of 25%.

The trial size of 36 patients for the primary endpoint is based on 80% power at a one-sided significance level of 5% to detect a difference of -20% in mean changes in tumour size at 24 weeks between placebo and cediranib, assuming CV of 25%.

20.8. Interim Analysis

A formal interim analysis is planned once 12 patients have been randomised to receive cediranib and 6 have been randomised to receive placebo, and these 18 patients have been followed up for 24 weeks (or until disease progression if sooner). The Independent Data Monitoring and Steering Committee (IDMSC) will advise on the exact timing of the interim analysis based on the number of unevaluble or ineligible patients entered into the trial (see Section 20.11). Recruitment to the study will not be halted while this interim analysis is conducted.

The randomisation aspect of the trial may be stopped at this stage on the basis of efficacy of cediranib if the one-sided p-value for the primary endpoint shows cediranib to be superior to placebo with p<0.01 (O'Brien-Fleming stopping rule). If this stopping rule is met, recruitment will continue to 36 patients but with all patients entering the trial after this interim analysis receiving cediranib from the outset. A p-value of 0.045 will be used for statistical significance in the final analysis to allow for this interim analysis.

20.9. Analysis Plan

For comparison of the percentage change in the sum of target marker lesion diameters at 24 weeks (or progression) between the cediranib and placebo groups, the Wilcoxon Mann Whitney test will be used. The primary analysis is planned when 36 patients have reached week 24 (or progression if sooner). The IDMSC will advise on the exact timing of the final analysis based on the number of unevaluable or ineligible patients entered into the trial (see Section 20.11).

For the secondary endpoints, RECIST v1.1 response rate at 24 weeks, best RECIST v1.1 response, best reduction (%) in tumour size and percentage alive and progression-free at 12 months will be reported with 95% confidence intervals. Progression free survival and overall survival will be characterized using Kaplan Meier curves. Toxicity and the frequency and nature of adverse events will be tabulated.

The analysis of the primary endpoint will be performed using all evaluable patients i.e., those who have a scan at 24 weeks or at disease progression if sooner. Further details of analysis populations and methods used will be described within the trial statistical analysis plan.

20.10. Endpoint Definitions

For the primary endpoint, the percentage change in the sum of the longest diameters of target marker lesions will be measured at 24 weeks from the date of randomisation if regression or stable disease according to RECIST v1.1 , or at the time of progression if this occurs before 24 weeks.

Progression free survival (PFS) will be measured from the date of randomisation until the first date of confirmed disease progression according to RECIST v1.1 or death (this is applicable for all patients whether they start on active or placebo treatment). This definition of PFS also applies to patients who are randomised to placebo, and then switch to cediranib at 24 weeks.

Best response/best reduction in tumour size of a patient will be defined as the best response/reduction in tumour size that is observed.

Response rate will be defined as the percentage of patients with complete response or partial response according to RECIST v1.1 at 24 weeks.

The percentage of patients alive and progression-free will be measured at 12 months from the date of randomisation. Patients will be defined as progression-free if regression or stable disease is confirmed according to RECIST v1.1.

Overall survival time will be measured from the date of randomisation until the date of death due to any cause. Time to last observation will be used if the patient has not died and survival time for the patient will be considered censored,

20.11. Independent Data Monitoring Committee

The replacement of patients who do not start treatment, withdraw from the study, are unevaluable for reasons deemed not to be treatment related or who are unevaluable for any other reason will be based on the advice of the IDMSC (see Section 21.2). The IDMSC will also advise on the exact timings of interim and final analyses based on the number of unevalub le or ineligible patients entered into the trial.

21. TRIAL MANAGEMENT

21.1. Trial Management Group (TMG)

A Trial Management Group (TMG) will be set up and will include the Chief Investigator, Co-investigators (including Sarcoma Group leads and country Lead Investigators) and identified collaborators, the Trial Statistician and the Trial Managers. Selected Principal Investigators and key study personnel will be invited to join the TMG as appropriate to ensure representation from a range of centres and professional groups. Notwithstanding the legal obligations of the Sponsor and Chief Investigator, the TMG have operational responsibility for the conduct of the trial. Where possible, membership will include a lay/consumer representative. The Committee's terms of reference, roles and responsibilities will be defined in a charter issued by ICR-CTSU.

21.2. Independent Data Monitoring and Steering Committee

A joint Independent Data Monitoring and Steering Committee (IDMSC) will be set up and will include an independent Chairman (not involved directly in the trial other than as a member of the IDMSC), not less than two other independent members. The Chief Investigator and one or two Principal Investigators may also attend the public aspects of the meetings. The trial statistician would also attend and supply the committee with all applicable data and information, for all discussions with the exception of private discussions of the committee.

It is the role of the IDMSC to monitor progress of the trial and safety of participants, and to ensure there is adherence to the protocol and the principles of Good Clinical Practice.

The Committee's terms of reference, roles and responsibilities will be defined in a charter issued by ICR-CTSU.

The Committee should meet in confidence at regular intervals, and at least annually. A report of the findings and recommendations will be produced following each meeting. This report will be submitted to the TMG, and if required, the relevant IECs and Competent Authorities.

Any final decision to stop the randomisation aspect of the trial at the halfway stage will be made by the IDMSC, guided by the interim analysis described in section 20.8.

The Committee reserve the right to release any data on outcome or side-effects to the TMG (and if appropriate to participants) if it determines at any stage that the combined evidence from this and other studies justifies it.

22. RESEARCH GOVERNANCE

22.1. Co-sponsor Responsibilities

The co-sponsors of this clinical trial are The Institute of Cancer Research (ICR) and the Royal Marsden Foundation Trust Hospital.

This Clinical Trial will be conducted in accordance with the ethical principles laid down by the Declaration of Helsinki, 1964 and as amended in 1996 and the principles of Good Clinical Practice.

This Clinical Trial will also be conducted in compliance with the trial protocol, all applicable international guidelines, and the national laws and regulations of the countries in which the Clinical Trial is performed. Sponsorship activities and delegated responsibilities are shared between the ICR and RMH. The responsibilities of the co-sponsors are set out in an agreement letter between ICR and RMH. Both parties agree to allow inspection of their premises by the competent authorities.

22.2. Sarcoma Group Responsibilities

Each participating Sarcoma Group will be responsible for country specific trial coordination including IEC / regulatory approvals and safety reporting. Exact responsibilities including translation of essential documents and country specific monitoring will be detailed in a contract between the co-sponsors and each individual Sarcoma Group.

22.3. Principal Investigator Responsibilities

Responsibilities of each Principal Investigator and participating centre will be detailed in a contract with the co-sponsors or with the country specific Sarcoma Group with trial coordination responsibilities in that country.

Principal Investigator Responsibilities:

Principal Investigator responsibilities include putting and keeping in place arrangements to run the trial at their site according to the trial protocol and applicable guidance notes, local regulations and the principles of GCP. The above responsibilities include, but are not limited to, ensuring that:

- the applicable ethical and institution specific approvals are in place before recruiting patients;
- sufficient data is recorded for all patients to enable accurate linkage between hospital records and CRFs;
- source data and all trial related documentation are accurate, complete, maintained and accessible for monitoring and audit visits;
- all staff involved with the trial are trained in and work to the applicable regulatory requirements;
- original consent forms are personally signed and dated by both the patient and investigator and are kept together in a central log together with a copy of the specific patient information sheet(s) given at the time of consent;
- all essential documents are retained in accordance with local regulations;
- staff comply with the protocol and Trial Guidance Notes for the trial.
- SAEs are reported to the ICR-CTSU within the timelines detailed above

Local Pharmacy Responsibilities:

The responsibilities of the designated responsible person within the pharmacy at each participating centre include, but are not limited to, ensuring that:

- study drug is handled and stored safely and according to product specific requirements;
- the study drug is dispensed only to trial patients and in accordance with the protocol;
- there is a sufficient supply of study drug for patients' continued treatment, and in a timely manner arrange for re-supply of stock;
- study drug expiry dates are monitored and drug is used in order of expiry date i.e. earliest expiry first;
- unused study drug is destroyed locally in accordance with local protocol;
- Study drug receipt, accountability and destruction records are maintained.

22.4. AstraZeneca Responsibilities

AstraZeneca are responsible on behalf of the co-sponsors for the manufacture, packing, labelling and distributing of study drug and matching placebo to site in accordance with Good Manufacturing Practice and all applicable local legislation. Some of these responsibilities have been delegated by AstraZeneca to Fisher Clinical Services. Responsibilities are defined in an agreement between AstraZeneca and the co-sponsors, and AstraZeneca and Fisher Clinical Services.

23. TRIAL ADMINISTRATION & LOGISTICS

23.1. Data Acquisition

ICR-CTSU is responsible for the central coordination of data management and statistical analysis of trial data. ICR-CTSU will supply paper CRFs to participating sites for the collection of trial specific data. The Trial Management Group reserves the right to amend or add to the CRF as appropriate. Such changes do not constitute a protocol amendment, and revised or additional forms should be used by centres in accordance with the guidelines provided by ICR-CTSU.

The clinical data should be reported on the CASPS case report forms (CRFs) to the ICR-CTSU in a timely manner. Specific guidance on how data will be collected will be detailed in trial guidance notes. On receipt at ICR-CTSU, CRFs will be recorded as received and any missing data will be reported to the originating site.

23.2. Central Data Monitoring

ICR-CTSU will review incoming CRFs for compliance with the protocol, and for inconsistent or missing data. Should any missing data or data anomalies be found, queries will be sent to the relevant centre for resolution. Following initial review, the CRF data items will be entered into the clinical study database held at ICR-CTSU.

Data will be further reviewed for data anomalies / missing data, by central statistical monitoring. Any systematic inconsistencies identified may trigger monitoring visits to centres.

23.3. On-Site Monitoring

If a monitoring visit is required, ICR-CTSU (or a representative from the national Sarcoma Group) will contact the centre to discuss dates of proposed visit. Once a date has been confirmed, the centre should ensure that the relevant patient source documents are available for monitoring.

If any problems are detected in the course of the monitoring visit, ICR-CTSU (or a representative from the national Sarcoma Group) will work with the Principal Investigator to resolve issues and, if necessary, to determine the centre's future participation in the study.

ICR-CTSU staff (or a representative from the national Sarcoma Group) conducting on-site monitoring will review essential documentation and carry out source data verification to confirm compliance with the site agreement and trial protocol, and to ensure the protection of patients' rights as detailed in the Declaration of Helsinki 1964 as amended October 1996.

23.4. Completion of the Study and Definition of Study End Date

According to applicable regulations, the study is deemed to have ended on the date of last data capture.

23.5. Archiving

Essential documents are those that individually and collectively permit evaluation of the conduct of the trial and substantiate the quality of the data collected. Essential documents will be maintained at ICR-CTSU in a way that will facilitate the management of the trial, audit and inspection. They will be retained for a sufficient period (at least 15 years) for possible audit. Documents will be securely stored and access restricted to authorised personnel.

Essential Documents should also be archived at each participating centre in accordance with local regulations.

24. PATIENT PROTECTION

This trial has been formally assessed for clinical risk using the ICR-CTSU risk assessment tool.

The trial will have received ethical, regulatory and institution specific approvals prior to recruitment of any patients into the study.

Patients will be asked to sign and date the trial consent forms after receiving both verbal and written information about the trial. All consent forms must be countersigned by the Principal Investigator or a designated individual. A record listing the designated individuals and the circumstances under which they may countersign consent forms must be clearly documented at the research site as part of the Delegation of Responsibilities Log. This log, together with original copies of all signed patient consent forms, must be available for inspection.

24.1. Patient Confidentiality

If applicable, patients will be asked to consent to their full name being collected at randomisation in addition to their date of birth. The personal data recorded on all documents will be regarded as confidential, and any information which would allow individual patients to be identified will not be released into the public domain.

Each investigator should keep a separate log of all patients' Trial IDs, names, addresses and hospital/healthcare numbers. The investigator must maintain trial documents, which

are to be held at the participating centres (e.g. patients' written consent forms), in strict confidence. The investigator must ensure the patients' confidentiality is maintained.

ICR-CTSU will maintain the confidentiality of all patient data received and will not reproduce or disclose any information by which patients could be identified. Representatives of ICR-CTSU (including those of local Sarcoma Groups in each country), the regulatory authorities, ethics committees and AstraZeneca may be required to have access to patients notes and study records for quality assurance purposes but patients should be reassured that their confidentiality will be respected at all times.

24.2. Data Protection

ICR-CTSU will comply with all applicable data protection laws. Any requests from patients for access to data about them held at ICR-CTSU should be directed to the Trial Manager in the first instance who will refer the request to the Data Protection Officer at The Institute of Cancer Research.

24.3. Liability and Insurance

The Co-Sponsors have taken out an insurance policy to cover their study responsibilities, and certification of this will be provided to the regulatory authorities as required. ICR-CTSU will need to be satisfied that all participating sites have appropriate indemnity arrangements in place.

25. FINANCIAL MATTERS

The trial is investigator designed and led and has been approved by the Clinical Trials Advisory & Awards Committee (CTAAC) of Cancer Research UK.

ICR has received funding from Cancer Research UK and AstraZeneca for the central coordination of the trial. AstraZeneca are also providing cediranib for the purposes of this trial free of charge.

In the UK, the trial meets the criteria for R&D support as outlined in the Statement of Partnership on Non-Commercial R&D in the NHS in England. The trial is part of the National Institute for Health Research (NIHR) portfolio by virtue of its approval by CTAAC. Therefore, NIHR Clinical Research Network (CRN) resources should be made available for the CASPS trial to cover UK specific research costs.

Country specific funding, if outside the UK, will be sourced and coordinated by the Sarcoma Group in each participating country.

26. PUBLICATION POLICY

The main trial results will be published in the name of the trial in a peer-reviewed journal, on behalf of all collaborators. The manuscript will be prepared by a writing group, appointed from amongst the Trial Management Group, and participating clinicians. All participating centres and clinicians will be acknowledged in this publication together with staff from the ICR-CTSU. All presentations and publications relating to the trial must be authorised by the Trial Management Group, on whose behalf publications should usually be made. Authorship of any secondary publications e.g. relating to the various biological studies will reflect the intellectual and time input into these studies, and will not be the same as on the primary publication. No investigator may present or attempt to publish data relating to the CASPS trial without prior permission from the Trial Management Group.

REFERENCES

1. Folpe AL, Deyrup AT: Alveolar soft-part sarcoma: a review and update. J Clin Pathol 59:1127-32, 2006

2. Reichardt P, Lindner T, Pink D, et al: Chemotherapy in alveolar soft part sarcomas. What do we know? Eur J Cancer 39:1511-6, 2003

3. Kayton ML, Meyers P, Wexler LH, et al: Clinical presentation, treatment, and outcome of alveolar soft part sarcoma in children, adolescents, and young adults. J Pediatr Surg 41:187-93, 2006

4. Lazar AJ, Das P, Tuvin D, et al: Angiogenesis-promoting gene patterns in alveolar soft part sarcoma. Clin Cancer Res 13:7314-21, 2007

5. Drevs J, Siegert P, Medinger M, et al: Phase I clinical study of AZD2171, an oral vascular endothelial growth factor signaling inhibitor, in patients with advanced solid tumors. J Clin Oncol 25:3045-54, 2007

6. Stacchiotti S, Tamborini E, Marrari A, et al: Response to sunitinib malate in advanced alveolar soft part sarcoma. Clin Cancer Res 15:1096-104, 2009

7. Gardner K JI, Leahy M, Barquin E, Marotti M, Collins B, Young H, Scurr M. : Activity of cediranib, a highly potent and selective VEGF signaling inhibitor, in alveolar soft part sarcoma. J Clin Oncol 27:Abstract 10523, 2009

8. Choi H, Charnsangavej C, Faria SC et al: Correlation of computed tomography and positron emission tomography in patients with metastatic gastrointestinal stromal tumour treated at a single institution with imatinib mesylate: proposal of new computed tomography response criteria. J Clin Oncol 2007; 25: 1753-1759.

9. Eisenhauer EA, Therasse P, Bogaerts J, et al: New response evaluation criteria in solid tumours: revised RECIST guideline (version 1.1). Eur J Cancer 45:228-47, 2009

APPENDIX 1: RECIST CRITERIA GUIDELINES

Response Evaluation Criteria in Solid Tumours (RECIST) Version 1.1 criteria should be used for the assessment of treatment outcomes.

Evaluation of measurable and non-measurable lesions

- **Measurable disease** – the presence of at least one measurable lesion. If the measurable disease is restricted to a solitary lesion, its neoplastic nature should be confirmed by cytology / histology.
- **Measurable lesions** – lesions that can be accurately measured in at least one dimension with the longest diameter ≥ 20 mm by chest X-ray, or ≥ 10 mm by CT/MRI scan or clinical exam.
- Lytic bone lesions or mixed lytic-blastic lesions, with identifiable soft tissue components, that can be evaluated by cross sectional imaging techniques such as CT or MRI can be considered as measurable lesions if the soft tissue component meets the definition of measurability described above.
- 'Cystic lesions' thought to represent cystic metastases can be considered as measurable lesions, if they meet the definition of measurability described above. However, if non-cystic lesions are present in the same patient, these are preferred for selection as target lesions.
- Malignant lymph nodes must be ≥15 mm in the short axis when assessed by CT scan to be considered measurable.
- **Non-measurable lesions** – all other lesions, including small lesions and malignant lymph nodes (longest diameter <10 mm, or pathological lymph nodes with ≥10 to <15 mm short axis) i.e., leptomeningeal disease, ascites, pleural/pericardial effusion, inflammatory breast disease, lymphangitis cutis/pulmonis, cystic lesions, blastic bone lesions and also abdominal masses that are not confirmed and followed by imaging techniques.
- Tumour lesions situated in a previously irradiated area, or in an area subjected to other loco-regional therapy, are usually not considered measurable unless there has been demonstrated progression in the lesion.
- The utilisation of endoscopy and laparoscopy for objective tumour evaluation is not advised. The utilisation of such techniques should be restricted to confirming complete pathological response when biopsies are obtained.

Baseline documentation of target and non-target lesions

- Measurable lesions up to a maximum of 2 lesions per organ and 5 lesions in total representative of all involved organs should be identified as target lesions and recorded and measured at baseline.
- Target lesions should be selected on the basis of their size (lesions with the longest diameter) and their suitability for accurate repeated measurements.
- All measurements should be taken and recorded in metric notation, using a ruler or calipers. All screening evaluations should be performed as closely as possible to the beginning of treatment and never more than 4 weeks before the beginning of treatment.
- The same method of assessment and the same technique should be used to characterise each identified and reported lesion at baseline and during follow up.
- A sum of the longest diameters (LD) for all target lesions will be calculated and reported as the baseline sum of LD. The baseline sum LD will be used as reference by which to characterise the objective tumour.
- All other lesions (or sites of disease) should be identified as non-target lesions and should also be recorded at baseline. Measurements of these lesions are not required, but the presence or absence of each should be noted throughout follow up. In addition, it is possible to record multiple non-target lesions involving the same organ as a single item on the case record form (e.g. 'multiple enlarged pelvic lymphnodes' or 'multiple liver metastases').

Response criteria

Documentation of new lesions

- The presence of a new lesion should be unequivocal: i.e. not attributable to differences in scanning technique, change in imaging modality or findings thought to represent something other than tumour (for example, some 'new' bone lesions may be simply healing or flare of pre-existing lesions).
- A lesion identified at a follow-up visit in an anatomical location that was not scanned at baseline is considered a new lesion and will indicate disease progression.

Lesions that become 'too small to measure'

- If lesions or lymph nodes recorded as target lesions at baseline become too faint on CT scan to assign an exact measure, a default value of 5mm should be assigned. This default value is derived from the 5mm CT slice thickness
- If it is the opinion of the radiologist that the lesion has likely disappeared, the measurement should be recorded as 0mm.

Evaluation of target lesions

Response criteria	Evaluation of target lesions
Complete Response (CR)	Disappearance of all target lesions (lymph nodes must be <10mm short axis)
Partial Response (PR)	At least 30% decrease in the sum of LD of target lesions, taking as reference the baseline sum of LD.
Progressive Disease (PD)	At least 20% increase in the sum of LD of target lesions, taking as reference the smallest sum LD recorded since the treatment started and at least 5mm absolute increase in this sum or the appearance of one or more new lesions.
Stable Disease (SD)	Neither sufficient shrinkage to qualify for PR nor sufficient increase to qualify for PD, taking as reference the smallest sum LD since the treatment started.

Evaluation of non-target lesions

Response criteria	Evaluation of non-target lesions
Complete Response (CR)	Disappearance of all non-target lesions
Incomplete response / Stable disease (SD)	Persistence of one or more non-target lesions
Progressive disease (PD)	Appearance of one or more new lesions and/or unequivocal progression of existing non-target lesions *

* To achieve 'unequivocal progression' on the basis of the non-target disease, there must be an overall level of substantial worsening in non-target disease such that, even in presence of SD or PR in target disease, the overall tumour burden has increased sufficiently to merit discontinuation of therapy. A modest 'increase' in the size of one or more non-target lesions is usually not sufficient to quality for unequivocal progression status. Although a clear progression of a non-target lesion is exceptional, in such circumstances, the opinion of the treating physician should prevail and the progression status should be confirmed later on by the review panel (or Chief Investigator).

Evaluation of overall response

The table below provides a summary of the overall response calculation at each time point.

Target lesions	Non-Target lesions	New Lesions	Overall response
CR	CR	No	CR
CR	Incomplete response/SD	No	PR
CR	Not-evaluated*	No	PR
PR	Non-PD or not all evaluated	No	PR
SD	Non-PD or not all evaluated	No	SD
Not all evaluated	Non-PD	No	Not evaluable
PD	Any	Yes or No	PD
Any	PD	Yes or No	PD
Any	Any	Yes	PD

* When no imaging/measurement is done at all at a particular time point, the patient is not evaluable (NE) at that time point. If only a subset of lesion measurements are made at an assessment, usually the case is also considered NE at that time point, unless a convincing argument can be made that the contribution of the individual missing lesion(s) would not change the assigned time point response.

Confirmation of disease progression

For equivocal findings of progression (e.g. very small and uncertain new lesions; cystic changes or necrosis in existing lesions), treatment may continue until the next scheduled assessment. If at the next scheduled assessment, progression is confirmed, the date of progression should be the earlier date when progression was suspected.

Patients with a global deterioration of health status requiring discontinuation of treatment without objective evidence of disease progression at that time should be classified as having "symptomatic deterioration". Every effort should be made to document the objective progression even after discontinuation of treatment.

In some circumstances it may be difficult to distinguish residual disease from normal tissue. When the evaluation of complete response depends on this determination, it is recommended that the residual lesion be investigated (fine needle aspirate/biopsy) to confirm the complete response status

Duration of overall response

The duration of overall response is measured from the time measurement criteria are met for CR or PR (whichever status is recorded first) until the first date that recurrence or PD is objectively documented, taking as reference for PD the smallest measurements recorded since the treatment started.

Duration of stable disease

SD is measured from the start of the treatment until the criteria for disease progression are met, taking as reference the smallest measurements recorded since the treatment started.

Evaluation of best overall response

The best overall response is the best response recorded from the start of the study treatment until disease progression. The patient's best overall response assignment will depend on the findings of both target and non-target disease and the appearance of new lesions.

Best overall response is defined as the best response across all time points. For example, a patient who has SD at first assessment, PR at second assessment, and PD on last assessment has a best overall response of PR.

APPENDIX 2: ECOG PERFORMANCE STATUS

ECOG PERFORMANCE STATUS	
**Grade**	**ECOG**
0	Fully active, able to carry on all pre-disease performance without restriction
1	Restricted in physically strenuous activity but ambulatory and able to carry out work of a light or sedentary nature, e.g., light house work, office work
2	Ambulatory and capable of all selfcare but unable to carry out any work activities. Up and about more than 50% of waking hours
3	Capable of only limited selfcare, confined to bed or chair more than 50% of waking hours
4	Completely disabled. Cannot carry on any selfcare. Totally confined to bed or chair
5	Dead

APPENDIX 3: HYPERTENSION MANAGEMENT GUIDELINES

For all BP thresholds described in Table 1, a trigger level is considered to be met if either the systolic and/or the diastolic pressure reach the threshold. If the threshold is recorded at home, it must be confirmed by a healthcare professional before commencing any treatment.

**Table 1**	**Hypertension management protocol for emergent hypertension**
**Hypertension severity**	**Actions**
**Mild to moderate hypertension:**<br><br>BP 140/90 mmHg on 2 consecutive occasions >24 hours apart<br><br>OR<br><br>Increase in diastolic pressure by ≥20 mmHg or to ≥100 mmHg or increase in systolic pressure to ≥150 mmHg	1. Repeat reading at least 1 hour later. If isolated increase, increase BP monitoring to twice weekly by health professional or daily home monitoring. **Continue all study medication at the same dose.**<br>2. If confirmed by second reading, **continue all study medication at the same dose** and initiate monotherapy with a long acting dihydropyridine calcium-channel blocker (eg, nifedipine, amlodipine, felodipine) at low dose.<br>• If calcium channel blocker is contraindicated, use selective β-blocker first line.<br>• Monitor BP daily by health professional until it stabilises.<br>3. If BP >140/90 mmHg after 24 hours INCREASE the first agent to the full dose and consider adding an additional agent in combination (eg, selective β-blocker, low dose combined alpha- and β-blocker, thiazide diuretic [angiotensin converting enzyme inhibitors or angiotensin receptor blockers if specific indication]).<br>4. If BP >140/90 mmHg after a further 24 hours, add an additional agent if the patient is only on one new agent or increase to full doses of the 2-drug combination.<br>5. If BP >160/95 mmHg and is static or increasing after a further 48 hours, temporarily stop study medication and continue anti-hypertensive therapy under close supervision.<br>6. Restart all study medication at the same dose (with maintenance anti-hypertensive therapy) when BP ≤140/90 mmHg. Monitor BP at least every 2 days by health professional until steady state is reached (7 days) and BP stabilised.<br>7. If BP increases to >160/95 mmHg, follow step 5 and restart all study medication but with the tablets at 1 dose lower than the starting dose when BP<140/90 mmHg.<br>8. If BP increases to >160/95 mmHg, follow step 5 and restart all study medication but with the tablets at 2 doses lower than the starting dose when BP<140/90 mmHg.<br>9. If BP increases to >160/95 mmHg after 2 dose reductions despite anti-hypertensive therapy, permanently stop all study medication.

Table 1	Hypertension management protocol for emergent hypertension
**Hypertension severity**	**Actions**
**Severe hypertension:**<br><br>Increase in diastolic pressure to ≥110 mmHg or increase in systolic pressure to ≥180 mmHg on 2 readings >1 hour apart	1. **Temporarily stop all study medication** and consider if hospitalisation is necessary.<br>2. Initiate treatment with a 2-drug combination including a calcium channel blocker licensed for use in severe hypertension, tailored to the patient's underlying conditions and previous anti-hypertension treatment.<br>3. If there is evidence of target-organ damage, intravenous therapy should be considered while continuing oral therapy.<br>4. Nitrates may adversely affect the therapeutic mechanism of action of cediranib but should be used if clinically indicated..<br>5. Restart all study medication but with the tablets at 1 dose lower than the starting dose (with maintenance antihypertensive therapy) when BP ≤140/90 mmHg. Monitor BP at least every 2 days by health professional until steady state is reached (7 days) and BP stabilised.<br>6. If BP >160/95 mmHg and is increasing after 48 hours, temporarily stop all study medication and continue anti-hypertensive therapy under close supervision.<br>7. Restart all study medication but with the tablets at 2 doses lower than starting dose (with maintenance antihypertensive therapy) when BP ≤140/90 mmHg. Monitor BP at least every 2 days by health professional until steady state is reached (7 days) and BP stabilised.<br>8. If BP increases to >160/95 mmHg after 2 dose reductions despite antihypertensive therapy, permanently stop all study medication.

When managing mild to moderate hypertension, the following principles should be noted:

- Cediranib may cause rapid increases in BP in some patients. Patients who are already on anti hypertensive agents may be at higher risk of developing moderate or severe hypertension. In such patients, care should be taken to ensure blood pressure is well controlled to <140/90mmHg prior to commencing cediranib. This may mean adding or increasing doses of calcium channel blockers prior to commencing cediranib.
- Calcium channel blockers are the first line agents of choice.
- If calcium channel blockers are contraindicated, ACE inhibitors; Angiotensin receptor antagonists or β-blockers are alternative agents.
- Increase anti-hypertensives to maximum doses and add additional agents as required.
- It is recommended that no more than 2 additional drugs are added in a 48 hour period before temporarily stopping cediranib.
- The following cautions and contraindications should be noted:
- Calcium channel blockers: use with caution in patients with tachyarrhythmias, aortic stenosis, unstable angina or congestive cardiac failure and may cause headache.

- Short-acting non-dihydropyridines such as diltiazem or verapamil should be avoided since they may precipitate abrupt fall in BP and increase risk of myocardial ischaemia, infarction or stroke.
- Beta blockers: contraindicated in patients with asthma, chronic obstructive pulmonary disease and A-V block; they should be used with caution in patients with peripheral vascular disease and glucose intolerance and may cause fatigue.
- If diuretics are to be used, thiazides rather than loop diuretics are recommended.
- A record of the management of hypertension will be maintained.

CTC Grade 3 should be assigned if hypertension is not controlled after 48 hours of anti-hypertensive therapy.
